# Srf destabilizes cellular identity by suppressing cell-type-specific gene expression programs

**DOI:** 10.1038/s41467-018-03748-1

**Published:** 2018-04-11

**Authors:** Takashi Ikeda, Takafusa Hikichi, Hisashi Miura, Hirofumi Shibata, Kanae Mitsunaga, Yosuke Yamada, Knut Woltjen, Kei Miyamoto, Ichiro Hiratani, Yasuhiro Yamada, Akitsu Hotta, Takuya Yamamoto, Keisuke Okita, Shinji Masui

**Affiliations:** 1 Department of Life Science Frontiers, Center for iPS Cell Research and Application (CiRA), Kyoto University, 53 Kawahara-cho, Shogoin, Sakyo-ku, Kyoto 606-8507 Japan; 2 RIKEN Center for Developmental Biology (CDB), 2-2-3 Minatojima-minamimachi, Chuo-ku, Kobe 650-0047 Japan; 3 Department of Diagnostic Pathology, Kyoto University Hospital, 54 Kawahara-cho, Shogoin, Sakyo-ku, Kyoto 606-8507 Japan; 4 Hakubi Center for Advanced Research, Kyoto University, Yoshida-honmachi, Sakyo-ku, Kyoto 606-8501 Japan; 5 Faculty of Biology-Oriented Science and Technology, Laboratory of Molecular Developmental Biology, Kindai University, 930 Nishimitani, Kinokawa-shi, Wakayama 649-6493 Japan; 6 Division of Stem Cell Pathology, Center for Experimental Medicine and Systems Biology, The Institute of Medical Science, The University of Tokyo, 4-6-1 Shirokanedai, Minato-ku, Tokyo 108-8639 Japan; 7 Institute for Integrated Cell-Material Sciences (WPI-iCeMS), Kyoto University, Kyoto, 606-8507 Japan; 8 AMED-CREST, AMED 1-7-1 Otemachi, Chiyodaku, Tokyo 100-0004 Japan; 9 CREST (Core Research for Evolutional Science and Technology), JST (Japan Science and Technology Agency), Honcho 4-1-8, Kawaguchi, Saitama 332-0012 Japan

## Abstract

Multicellular organisms consist of multiple cell types. The identity of these cells is primarily maintained by cell-type-specific gene expression programs; however, mechanisms that suppress these programs are poorly defined. Here we show that serum response factor (Srf), a transcription factor that is activated by various extracellular stimuli, can repress cell-type-specific genes and promote cellular reprogramming to pluripotency. Manipulations that decrease β-actin monomer quantity result in the nuclear accumulation of Mkl1 and the activation of Srf, which downregulate cell-type-specific genes and alter the epigenetics of regulatory regions and chromatin organization. Mice overexpressing *Srf* exhibit various pathologies including an ulcerative colitis-like symptom and a metaplasia-like phenotype in the pancreas. Our results demonstrate an unexpected function of Srf via a mechanism by which extracellular stimuli actively destabilize cell identity and suggest Srf involvement in a wide range of diseases.

## Abstract (web summary)

The transcription factor Srf is a central regulator of immediate-early and actin cytoskeletal genes. Here the authors show that Srf is activated by repression of β-actin, promoting iPSC reprogramming of neural progenitor cells and hepatoblasts by repressing cell-type specific genes.

## Introduction

All cell types in an organism are generated through a number of differentiation events that involve the loss of one cell identity for another. The maintenance of cell identity is crucial for organismal homeostasis and a loss of this maintenance is associated with aging and diseases such as cancer^1,2^. How cell identity is regulated is thus a fundamental biological question.

Cell identification is regulated by specific gene expression programs. Extracellular signals such as growth factors, extracellular matrices, and their stiffness are received by specific receptors that transduce the signals intracellularly^3^, to regulate the activity of transcription factors (TFs)^4^. TFs regulate gene expressions for which regulatory elements including enhancers and promoters are essential^4^. Master TFs regulate gene expressions that are specific for cell identity by binding to many enhancers, including super-enhancers, which encompass large regions and have stronger activity^5^. Master TFs form a core transcriptional network that primarily maintains the gene expression program specific for the cell type^6^. Indeed, the ectopic expression of master TFs can change the fate of somatic cells to other cell types^7^. One of the most well-known examples of cell fate change is the reprogramming of cells into induced pluripotent stem cells (iPSCs), which have a potency equivalent to embryonic stem cells (ESCs), by the overexpression of the master TFs for ESCs (*Oct4*, *Klf4*, *c-Myc*, and *Sox2* (OKMS)) in somatic cells^8^. Yet, how master TFs maintain cell identity remains unclear, presumably due to the fact that many crucial molecules and pathways involved in the maintenance of cell identity are still unknown. Reprogramming to iPSCs is one way to find these molecules and pathways.

Reprogramming needs to pass through several molecular pathways and the genes involved in these pathways can be identified by screenings^9,10^. Accordingly, many factors have been reported as “roadblocks” of reprogramming and presumably maintain somatic cell identity^9,10^. However, the majority of these factors have been studied only in one specific cell type (typically fibroblasts), despite the fact that functional differences in roadblock factors depend on cell type^11^. To study cell-type-specific mechanisms for cell identify maintenance, here we sought to identify roadblock genes in two diverse cell types, neural and liver cells. Knockdown screenings identify many cell-type-specific genes in each cell type as well as ubiquitous genes including the β-actin gene and genes involved in β-actin dynamics. The manipulation of β-actin dynamics activates serum response factor (Srf) through the canonical pathway^12^, which unexpectedly downregulates cell-type-specific genes through direct binding, at least partially. Misactivation of Srf in mice induces various pathologies that have been associated with super-enhancers responsible for maintaining cell identity. As Srf is activated by a variety of extracellular signals^13–16^, our data indicate that Srf can destabilize cell identity in response to exogenous cues in broad cell types and suggest that Srf misactivation could be a novel mechanism for the induction of various diseases.

## Results

### Cell-type-specific genes maintain cell identity

**Fig. 1:**
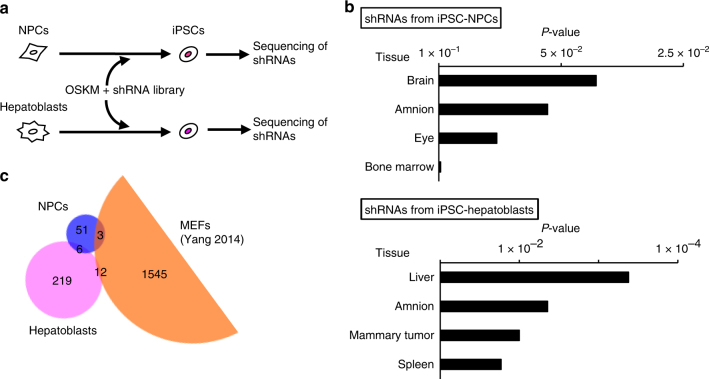
Cell-type-specific genes inhibit reprogramming. **a** Outline of the screening strategy used to identify factors that inhibit reprogramming. **b** Cell-type-specific genes are enriched by the screenings. Genes enriched in the second screening of NPCs and the high positive fraction in the screening of hepatoblasts were subjected to tissue expression analysis with DAVID^18, 19^. Fisher’s exact test. **c** Factors that inhibit reprogramming are different among cell types. Identified genes from the second screening of NPCs, the high positive fraction in the screening of hepatoblasts and Yang et al.^20^ were used to generate the Venn diagram. Numbers indicate the number of genes

To identify the factors involved in the maintenance of cellular identity, we used a well-studied system that reprograms somatic cells into iPSCs^8^. To identify inhibitory factors for cell reprogramming (i.e., factors important for the maintenance of somatic cell identity), short hairpin RNA (shRNA)-based knockdown library screenings were performed using a reprogramming system of neural progenitor cells (NPCs) as a model (Supplementary Fig. 1). The NPCs were generated by in vitro differentiation of mouse ESCs and maintained in a two-dimensional culture condition^17^. Later, we introduced into them a cocktail of the reprogramming factors OKMS, inducible under the control of a doxycycline (Dox)-inducible promoter. In addition, the NPCs carry a blasticidin-resistant gene under the control of the endogenous *Oct4* allele for the selection of reprogrammed iPSCs. We also established a “suboptimal” concentration of Dox (40 ng/ml) that failed to induce cellular reprogramming, to create a meta-stable cellular state that enhances sensitivity to a shRNA screening for the identification of factors associated with the maintenance of cellular identity. Accordingly, at 40 ng/ml Dox, no colonies were formed and no cells survived when selected for the *Oct4*-blasticidin reporter. We then applied a genome-wide shRNA library at this Dox concentration into the NPCs. No cells survived when shLuc was introduced (negative control), but iPSC colonies resistant to blasticidin emerged when the shRNA library was introduced. To identify candidate shRNAs, we collected DNA from these iPSCs, recovered shRNA sequences by PCR, and analyzed the sequences by deep sequencing (Fig. 1a and Supplementary Fig. 1). To further enrich shRNAs associated with the promotion of reprogramming, we introduced a second library, which was prepared using shRNA fragments amplified from genomic DNA from the iPSCs in the first screening, and repeated the analysis. The enrichment of each shRNA sequence compared with total sequence reads was calculated for the first and second screenings. As a result, 1544 and 60 shRNA sequences were enriched ( > 1-fold), respectively (Supplementary Data 1). The reliability of the screenings was confirmed by experiments in which shRNAs from the second screening were individually checked for the promotion of reprogramming (Supplementary Fig. 2a). We analyzed target genes using a database for expression specificity across tissues^18,19^ and found that the shRNA targets after the screenings were enriched with genes expressed in the brain, which is consistent with the use of neuronal lineage cells as the iPSC origin (Fig. 1b and Supplementary Figs. 2b and 3a). In addition, genes more highly expressed in NPCs ( > 2-fold) compared with those in ESCs (designated as “NPC genes”) were enriched (Supplementary Fig. 3b). These results suggest that knockdown of cell-type-specific genes accelerates reprogramming, and that these genes tend to maintain the cell identity of NPCs.

To clarify whether these results with NPCs, which are of ectoderm origin, are also applicable to other lineages, we used mouse hepatoblasts harboring a BAC transgene of Nanog-EGFP^11^ (Fig. 1a and Supplementary Fig. 4a, b). These cells are of endoderm origin and were isolated from fetal liver in primary culture. Consistent with the NPC results, genes expressed in liver were enriched in shRNA targets in efficiently reprogrammed cells from hepatoblasts (Fig. 1b and Supplementary Data 2). In addition, we found that a relatively small number of shRNA targets in NPCs, hepatoblasts, and mouse embryonic fibroblasts^20^ overlapped with one another (Fig. 1c, Supplementary Fig. 4c and Supplementary Data 3). Although representative inhibitory genes such as growth suppressor *p53*^21^ (also known as *Tp53*) and epigenetic repressor *Mbd3*^22^ were not found in shRNA targets in NPCs or hepatoblasts, we found *Cdk2ap1*, which negatively regulates proliferation^23^, and *Dnmt3b*, which confers repressive epigenetic modifications^24^ (Supplementary Data 1 and 2), suggesting that pathways with similar functions might inhibit reprogramming but that the contribution of individual genes may depend on the cell type^25^. Another representative pathway in reprogramming is the mesenchymal-to-epithelial transition (MET), in which mesenchymal and epithelial genes are down- and upregulated, respectively^26,27^. NPCs express mesenchymal genes^11^, whereas hepatoblasts express epithelial genes^11^. Accordingly, the identified shRNA targets in NPCs, but not those in hepatoblasts, could contain genes involved in the regulation of MET. An in silico analysis using the Ingenuity Pathway Analysis (IPA) showed a significant enrichment of genes related to “Regulation of the Epithelial-Mesenchymal Transition Pathway” in the first screening of NPCs (*P* = 0.0036, Fisher’s exact test), but no hits of MET/EMT-related terms in the screening of hepatoblasts, suggesting that the importance of MET for reprogramming may depend on cell type. Taken together, and consistent with a previous report in which cell-type-specific master TFs inhibited reprogramming^11^, our results suggest that the repression of cell-type-specific genes promoted reprogramming.

### β-Actin repression promotes reprogramming

**Fig. 2:**
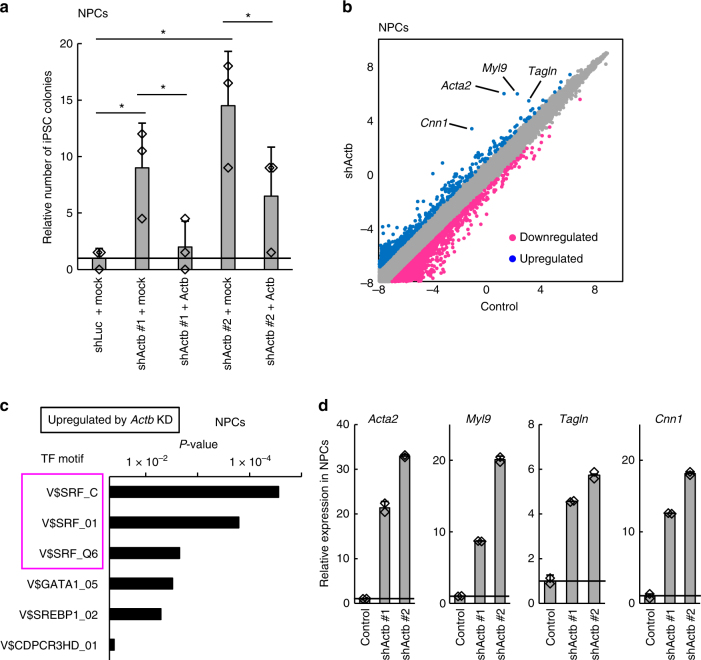
β-Actin inhibits reprogramming and Srf activity. **a** Knockdown of *Actb* gene expression enhances reprogramming. Reprogramming efficiencies from NPCs are shown as iPSC colony numbers relative to shLuc + mock-introduced cells. Means ± SD are shown (*n* = 3). Dots indicate individual data points. Student’s *t*-test (*P < 0.05). **b** Knockdown of *Actb* alters various gene expressions. A scatter plot comparing gene expressions of *Actb*-knockdown NPCs and control cells. Blue dots and magenta dots show genes upregulated and downregulated > 2-fold, respectively. Some known Srf target gene symbols are indicated. **c** Srf-binding consensus motifs are enriched upstream of genes upregulated by *Actb* knockdown. Genes upregulated > 2-fold in NPCs were subjected to a motif search using Transfac to search protein-binding consensus sequences that were enriched in upstream regions. **d** Srf is activated by *Actb* knockdown. Expression levels relative to control cells for each Srf target gene in NPCs are shown (*n* = 2, technical duplicate). Values are means ± SD of the microarray data. Dots indicate individual data points

Although the shRNA targets from the screenings contained many cell-type-specific genes, we also found some genes that are ubiquitously and highly expressed, such as the β-actin gene *Actb* (Supplementary Data 1). Therefore, we investigated *Actb* function in reprogramming. We first confirmed the effect of shRNAs for *Actb* (shActb) on reprogramming by using two different shRNA sequences to exclude the possibility of off-target effects. Both shActb introduced by retrovirus vectors effectively repressed *Actb* expression (Supplementary Fig. 5a). Introduction of these shActb into NPCs enhanced the production of iPSCs by Dox addition, as judged by *Oct4* expression, alkaline phosphatase expression, and 2i resistance (Fig. 2a). Such an effect was attenuated by the co-introduction of *Actb* (Fig. 2a). Together, these results indicate that *Actb* repression promoted reprogramming. We also confirmed that iPSCs derived with shActb retrovirus were able to contribute to various tissues such as neurons, cartilage, and digestive tissues in chimeric mice, suggesting their pluripotency (Supplementary Fig. 5b). In addition, although reprogramming can be enhanced by the acceleration of cell proliferation^28^, *Actb* knockdown had little effect on cell proliferation (Supplementary Fig. 5c), but it did elevate the reprogramming rate (Supplementary Fig. 5d), suggesting that *Actb* repression accelerates the dedifferentiation of NPCs to promote reprogramming. We further address the consequences of *Actb* repression later.

We also confirmed the effect of *Actb* repression in hepatoblasts and a ureteric bud cell (UBC) line, which is of mesoderm origin^29^. As knockdown by shActb was weak in hepatoblasts (Supplementary Fig. 6a), we also applied the CRISPR/Cas9 system to repress *Actb* expression in hepatoblasts (Supplementary Fig. 6b). The repression of *Actb* promoted reprogramming in both cell types (Supplementary Fig. 6c, d), suggesting that β-actin inhibits reprogramming during iPSC induction from cell types across different germ layers.

### Srf promotes reprogramming downstream of β-actin

**Fig. 3:**
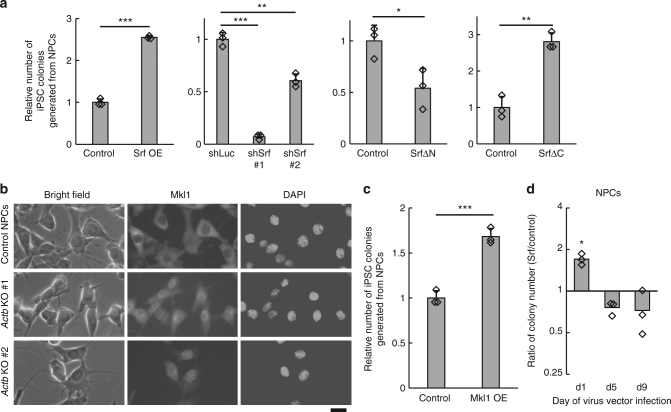
Srf and its cofactor, Mkl1, promote reprogramming. **a** Srf promotes reprogramming. Values are means ± SD of the numbers of iPSC colonies produced relative to control cells (*n* = 3). OE indicates overexpression. Dots indicate individual data points. Student’s *t*-test (***P < 0.0005, **P < 0.005, *P < 0.05). **b** Mkl1 mainly localizes in the nucleus upon β-actin depletion. Immunofluorescence microscopy of NPCs using anti-Mkl1 antibody and images of the corresponding bright field and DAPI staining. Bar, 20 µm. **c** Mkl1 promotes reprogramming. Values are means ± SD of numbers of iPSC colonies generated from NPCs relative to the control (*n* = 3). Dots indicate individual data points. Student’s *t*-test (***P < 0.0005). **d** Srf promotes reprogramming at early phase. NPCs were infected with *egfp*-expressing lentivirus or *Srf*-overexpressing lentivirus at the days indicated (d1 is the day of reprogramming initiation by Dox addition). Vertical axis shows the reprogramming efficiency of *Srf*-overexpressing cells to *egfp*-expressing cells in logarithmic (log) scale (*n* = 3). Dots indicate individual data points. Asterisks indicate statistically significant differences between *Srf* overexpression and control on the day of virus infection (Student’s *t*-test; *P < 0.05)

To identify the downstream targets of β-actin for transcriptional regulation, we analyzed the global gene expression of NPCs with shActb (Fig. 2b). In Gene Ontology (GO) analysis, downregulated genes included those for neural functions and development (Table 1). On the other hand, upregulated genes included those for cytoskeleton and cell motility (Table 1), and the upstream regions of these genes tended to contain consensus binding motifs of Srf (Fig. 2c). Indeed, known Srf targets such as *Acta2*, *Tagln*, *Myl9*, and *Cnn1* were upregulated by *Actb* repression (Fig. 2b, d), suggesting that β-actin depletion resulted in Srf activation (see below), as previously reported for actin polymerization^12^. Consistently, *Srf* overexpression also promoted reprogramming (Fig. 3a) without accelerating cell proliferation (Supplementary Fig. 7a), whereas knockdown of *Srf* suppressed reprogramming (Fig. 3a and Supplementary Fig. 7b). Reprogramming was also suppressed by the introduction of SrfΔN, which lacked the N-terminal region that contained the MADS box for DNA and cofactor binding^30^ (Fig. 3a and Supplementary Fig. 7c). On the other hand, another truncation mutant, SrfΔC, which lacked the C terminus but contained the MADS box, promoted reprogramming (Fig. 3a and Supplementary Fig. 7c). These results indicate that Srf promotes reprogramming through the MADS-box-containing N-terminal region, presumably due to its DNA- and/or cofactor-binding function. To address whether the function of Srf in NPCs are conserved in other cell lineages, hepatoblasts and UBCs were investigated. We found that Srf promoted reprogramming in these cell types too (Supplementary Fig. 7d, e), suggesting that the effect of Srf on reprogramming is common in a broad range of cell types.

**Table 1:** Representative GO terms for genes downregulated and upregulated by *Actb* knockdown

GO term	*P*-value
Downregulated	
Multicellular organismal process	9.06E–6
Animal organ development	4.10E–3
Regulation of release of sequestered calcium ion into cytosol	1.12E–2
Regulation of transmembrane transport	1.25E–2
Upregulated	
Contractile fiber	7.10E–4
Myofibril	3.41E–3
Sarcomere	1.10E–2
Muscle contraction	1.10E–2

Fisher’s exact test

GO, Gene Ontology

### Depletion of β-actin activates Mkl1-Srf pathway

We next addressed how β-actin regulated Srf activity. The polymerization dynamics of β-actin is known to regulate Srf activity through a pathway mediated by the nuclear-cytoplasmic shuttling of megakaryoblastic leukemia 1 (Mkl1, also known as MRTF-A or Mal), a cofactor of Srf^12^. The nuclear localization of Mkl1 is controlled by binding with G-actin (β-actin monomer); Mkl1 is exported to the cytoplasm when it binds to G-actin, but it is localized in the nucleus and can activate Srf when released from G-actin^12^. Hence, the nuclear localization of Mkl1 indicates a low abundance of G-actin within a cell. Immunocytochemistry of Mkl1 revealed that although Mkl1 was predominantly localized in the cytoplasm of control cells, it was more abundant in the nucleus when β-actin was depleted (Fig. 3b and Supplementary Fig. 8a, b), as seen in the case of actin polymerization^12^. In addition, transient treatment with jasplakinolide, a β-actin-polymerizing compound, which increases the nuclear localization of Mkl1 and activation of Srf^12^, promoted the early phase of reprogramming (Supplementary Fig. 8c). In contrast, treatment with latrunculin A, a β-actin-depolymerizing compound, suppressed reprogramming (Supplementary Fig. 8c). Moreover, *Mkl1* overexpression promoted reprogramming (Fig. 3c), whereas *Mkl1* and *Srf* knockdown nullified the enhanced reprogramming efficiency caused by β-actin depletion (Supplementary Fig. 8d). In contrast, the overexpression of *Elk3* or *Elk4*, ternary complex factors (TCFs) of another known Srf-activating pathway, repressed reprogramming (Supplementary Fig. 8e). As TCFs compete with Mkl1 for Srf binding^31^, this result suggests that TCFs might decrease the binding of Mkl1 to Srf, thus weakening the promotion of reprogramming. These results collectively suggest that Srf promotes reprogramming by signaling the G-actin pool depletion to downstream genes through Mk11.

### Srf preferentially downregulates cell-type-specific genes

Next, we explored the function of Srf in reprogramming. Reprogramming from somatic cells to iPSCs involves an early (initiation) phase, intermediate phase, and late (maturation and stabilization) phase, and different factors and pathways are involved in each phase^9,27^. To determine in which phase Srf promotes reprogramming, we overexpressed *Srf* by lentiviral transduction in NPCs at different time points. Interestingly, reprogramming was enhanced only when *Srf* was overexpressed in the early phase (Fig. 3d), a stage that is involved with the loss of somatic cell identity before *Oct4* is upregulated^9,11^. A similar result was observed in hepatoblasts (Supplementary Fig. 9a) and UBCs (Supplementary Fig. 9b), suggesting the conservation of Srf function beyond germ layers.

**Fig. 4:**
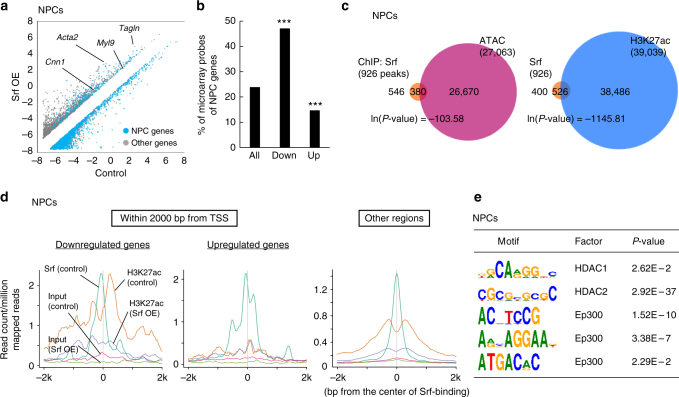
Srf preferentially downregulates cell-type-specific genes. **a** A scatter plot showing gene expressions that are > 2-fold different between *Srf*-overexpressing NPCs and control cells. Blue dots show NPC genes, which are defined as genes whose expression levels in NPCs are > 2-fold compared with those in ESCs. **b** Cell-type-specific genes are enriched in genes downregulated by *Srf* overexpression. Asterisks indicate a significant difference between genes in the indicated group and all other genes (two-sided Fisher’s exact test; ***P < 0.0005). **c** Srf preferentially binds to open and H3K27ac-marked genomic regions. The peak number in the overlapped area is based on the number of Srf-binding peaks. Overlaps were analyzed by the hypergeometric test. **d** Srf removes adjacent H3K27ac in NPCs. Means of ChIP-seq signals around genomic regions to which Srf was bound within 2000 bp from TSS (left panels) and other regions (right panel). **e** Matrices of consensus binding motifs of HDACs and Ep300 are enriched near Srf-binding sites. Motif analyses of ChIP-seq data of Srf in NPCs indicated that matrices of the motifs of HDAC1, HDAC2, and Ep300 were enriched within 25 bp of the Srf-binding sites in NPCs. Fisher’s exact test

To investigate this function in more detail, we analyzed global gene expressions in *Srf*-overexpressing NPCs. Noting that Srf targets mediated by Mkl1 were upregulated by *Srf* overexpression (Fig. 4a and Supplementary Fig. 10a, b) and changes in gene expressions caused by *Srf* overexpression resemble those caused by *Actb* knockdown and *Mkl1* overexpression (Supplementary Fig. 10c), overexpression of Srf roughly mimicked the activation of Srf. As the localization of Mkl1 was not altered by *Srf* overexpression (Supplementary Fig. 10d), we concluded that the upregulation of Mkl1-Srf target genes was due to the increased proportion of Mkl1 that interacted with Srf in the nucleus and not an increased nuclear import of Mkl1. We classified genes into three groups: “NPC genes,” which are more highly expressed in NPCs ( > 2-fold) than in ESCs; “ESC genes,” which are more highly expressed in ESCs ( > 2-fold) than in NPCs; and “ubiquitous genes,” which are specific neither for NPCs nor ESCs. The percentages of “NPC genes,” “ESC genes,” and “ubiquitous genes” compared with all genes were 23.8%, 32.5%, and 43.8%, respectively. Microarray analyses showed that almost half (47.0%) of the genes downregulated by *Srf* overexpression were NPC genes, indicating a significant number of cell-type-specific genes are suppressed by Srf when compared with genes that were not downregulated (*P* = 1.69 × 10^–299^, Fisher’s exact test) (Fig. 4a, b). On the other hand, only 14.6% of genes upregulated by *Srf* overexpression were NPC genes (Fig. 4a, b). The NPC genes downregulated by *Srf* overexpression tended to also be downregulated by shActb or *Mkl1* overexpression (Supplementary Fig. 10c), supporting the notion that β-actin acted through Mkl1 and Srf. These results are consistent with previous reports that showed Srf acts as a repressor as well as an activator^32^. GO terms of the genes downregulated by *Srf* overexpression included neuron-related terms with high significance (Table 2), whereas GO terms of genes upregulated by *Srf* overexpression included known functions of Srf such as mesoderm formation, cell adhesion, and motility^33^ (Table 2). We also analyzed the repression of cell-type-specific genes by Srf in hepatoblasts. As in NPCs, genes more highly expressed in hepatoblasts ( > 3-fold) than in ESCs were designated as “hepatoblast genes.” The proportion of hepatoblast genes in the downregulated genes by *Srf* overexpression ( > 1.5-fold) was 32.9%, indicating an enrichment of hepatoblast genes when compared with genes that were not downregulated (*P* = 8.31 × 10^–94^, Fisher’s exact test) (Supplementary Fig. 10e). These data suggest that Srf preferentially downregulates the expression of cell-type-specific genes and that Srf activation, either by G-actin reduction or *Srf* overexpression, promotes the loss of original cell identity.

**Table 2:** Representative GO terms for genes downregulated or upregulated by *Srf* overexpression

GO term	*P*-value
Downregulated	
Olfactory receptor activity	2.16E–23
Sensory perception of chemical stimulus	7.97E–23
Neurological system process	8.29E–22
G-protein coupled receptor protein signaling pathway	1.20E–20
Upregulated	
Blood vessel morphogenesis	2.84E–6
Pattern specification process	8.11E–6
Regulation of cell migration	5.20E–4
Biological adhesion	6.39E–3

Fisher’s exact test

GO, Gene Ontology

### Srf removes active enhancer/promoter marks

To analyze the mechanism by which Srf repressed cell-type-specific genes, chromatin immunoprecipitation sequencing (ChIP-seq) using Srf antibody was performed to map genome-wide Srf-binding sites in NPCs. We also performed ChIP-seq for the active enhancer/promoter mark H3K27ac and ATAC-seq analysis for open chromatin regions in NPCs. These analyses revealed that Srf-binding sites significantly overlapped with open chromatin regions and active enhancer/promoter regions (Fig. 4c). Indeed, the intensity of the ChIP-seq signals of H3K27ac correlated with those of Srf (Fig. 4d). We found that *Srf* overexpression decreased H3K27ac levels around Srf-binding sites in the putative promoter region (within 2000 bp from the transcription start site (TSS)) of downregulated genes and other regions (Fig. 4d). We further found that overexpression of *Mkl1*, which could activate Srf (Supplementary Fig. 10c), preferentially downregulated cell-type-specific genes (Supplementary Fig. 10f) similar to *Srf* overexpression (Fig. 4b). H3K27ac levels around the Srf-binding sites were also decreased by the overexpression of *Mkl1* near the TSS of downregulated cell-type-specific genes (Supplementary Fig. 11a). Furthermore, the whole amount of H3K27ac was comparable between *Srf*-overexpressing cells and control cells as estimated by western blotting analysis (Supplementary Fig. 11b, c), suggesting that Srf activity can inactivate adjacent genomic regions. As the removal of H3K27ac was not found around Srf near the TSS of upregulated genes (Fig. 4d), controlling H3K27ac levels at promoters as well as remote sites^31,32^ could be a mechanism by which Srf downregulates target genes. We found an enrichment of motifs for histone deacetylases (HDACs), which are involved in the inactivation of gene expression, near the Srf-binding sites in addition to an enrichment of binding motifs for Ep300 (also known as p300), which is required for enhancer activation (Fig. 4e). Similarly, in hepatoblasts, Srf-binding regions significantly overlapped with H3K27ac (Supplementary Fig. 11d) and the motifs of HDACs were enriched near the Srf-binding sites (Supplementary Fig. 11e). This observation is consistent with previous reports that demonstrated associations of HDACs with Srf^34,35^. Overall, these results suggest that Srf removes histone acetylation marks from active enhancers/promoters, possibly through its cooperation with HDACs.

### Srf activation leads to changes of subnuclear organization

**Fig. 5:**
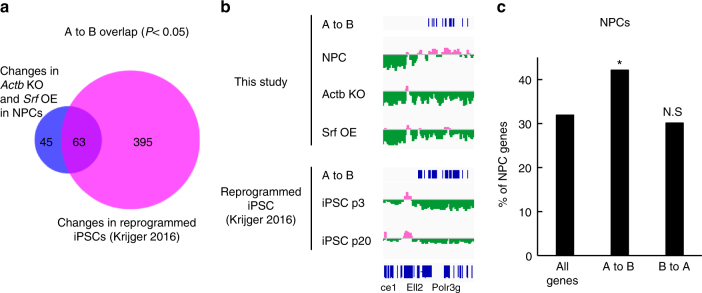
β-Actin-Srf pathway induces a global reorganization of the subnuclear compartment. **a** Activation of the β-actin-Srf pathway alone induces a global reorganization of the subnuclear compartment that resembles partial reprogramming. Venn diagram of the chromatin-organization changes from compartment A to compartment B in NPCs. Peak numbers are shown. **b** A region on chromosome 13 where the patterns from A to B are similar in *Actb* knockout, *Srf* overexpression in NPCs, and reprogramming to iPSCs. **c** Cell-type-specific genes are enriched in genes that change their subnuclear compartment from A to B by the β-actin-Srf pathway. Asterisks and N.S. indicate a significant difference and no significant difference, respectively, between genes in the indicated group and all other genes (two-sided Fisher’s exact test; *P < 0.05)

The subnuclear organization of the genome is closely associated with gene expression and differentiation states^36^. We thus addressed the effects of the β-actin-Srf pathway on the nuclear genome organization by performing Hi-C analyses using β-actin-depleted or *Srf*-overexpressing NPCs. By principal component analysis (PCA) of the Hi-C data, one can map distinct subnuclear compartments, A and B, on the genome, between interactions that are much less frequent than those within each compartment^37^. Compartment A corresponds to the accessible, active, and early-replicating euchromatin located in the interior of the nucleus, whereas compartment B corresponds to the inaccessible, inactive, and late-replicating heterochromatin located close to the nuclear envelope^37^. In general, the distribution of compartments A and B along the genome is considered cell-type specific^38^. PCA of the Hi-C data revealed that A-to-B switching regions by both β-actin depletion and *Srf* overexpression (without OKMS induction) overlapped well with those regions observed after reprogramming to iPSCs^39^ (Fig. 5a, b). Moreover, cell-type-specific genes were enriched in A-to-B switching regions, but not in B-to-A switching regions (Fig. 5c). However, statistical analyses showed no significance between Srf-binding sites and compartment changes (hypergeometric test). The insignificance suggests that Srf indirectly changes the 3D genome structure and/or a small subpopulation of Srf-binding sites are critical for the changes. Our data nevertheless suggest that Srf destabilizes cell-type specificity, partly through changes in the organization of subnuclear chromatin compartments, to promote the downregulation of cell-type-specific genes. Clarifying exactly how the compartments are changed by the β-actin-Srf pathway is an interesting future question. In contrast to cell-type-specific genes, ESC genes were not enriched in B-to-A switching regions by both β-actin depletion and *Srf* overexpression (Supplementary Fig. 12a). These genes were overlapped with the regions observed after reprogramming to iPSCs^39^ (Supplementary Fig. 12b, c), suggesting that the reprogramming promoted by the β-actin-Srf pathway may involve the priming of chromatin domains that contain non-ESC genes for activation in addition to the domain-level suppression of NPC genes.

### Extracellular stimuli control cell-type genes through Srf

**Fig. 6:**
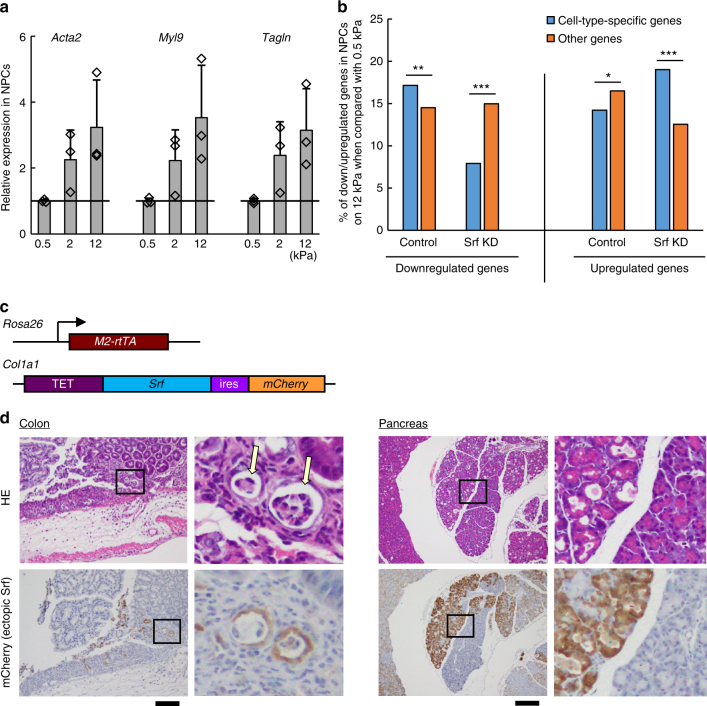
Srf misactivation causes various diseases. **a** Srf activity is regulated by an exogenous cue, extracellular matrix stiffness. The expressions of Srf target genes in NPCs cultured on matrices of various stiffness relative to the value of each gene on the softest matrix (0.5 kPa). Values are means ± SD of the relative expression levels normalized to *Gapdh* expression (*n* = 3). Dots indicate individual data points. **b** Cell-type-specific genes are downregulated by Srf with stiffness. Percentages of genes differentially regulated in NPCs among cell-type-specific genes and other genes between 0.5 and 12 kPa matrices. Two-sided Fisher’s exact test (***P < 0.0005, **P < 0.005, *P < 0.05). **c** Structure of transgenes in KH2-Srf mice. **d** Srf misactivation causes diseases. Immunohistological observations of chimeric mice that were treated with Dox for 7 days. Upper images are representative images of hematoxylin/eosin staining (HE) and lower images are roughly corresponding view fields of immunohistochemistry sections for *Srf* ectopic expression (mCherry) in colon and pancreas in a chimeric mouse overexpressing *Srf*. Right panels of each tissue are magnified views of the areas indicated by the squares in the respective left panels. Arrows indicate sites exhibiting crypt abscess. Bars, 200 µm

We next investigated the activity of Srf to repress cell-type-specific genes in response to extracellular mechanical stimuli. Srf is activated when cells are on a stiffer matrix through changed actin dynamics^13,40^. We thus examined whether the stiffness of the extracellular matrix contributes to the maintenance of the cell-type-specific gene expression program. We compared NPC cultures on a matrix of 0.5 kPa, which is a stiffness close to that of brain (soft; i.e., the physiological microenvironment of NPCs), and 12 kPa (hard). The activity of Srf exhibited a positive correlation with the stiffness, as judged by the expression of Mkl1-mediated Srf targets such as *Acta2* (Fig. 6a), consistent with a previous report^40^. In contrast, genes that qualified as cell-type-specific genes on the 0.5 kPa matrix were significantly downregulated in cells on the 12 kPa matrix (Fig. 6b). As each cell type resides in the specific stiffness of the surrounding microenvironment in vivo^13,40^, these results suggest that Srf is able to downregulate cell-type-specific gene expressions in response to deviations from optimal stiffness and that extracellular stimuli can suppress cell identity by activating Srf.

### Srf is a risk factor for various diseases

To address the importance of Srf activity on the maintenance of cell identity and homeostasis of an organism, we generated mice in which *Srf* could be overexpressed by the administration of Dox. We introduced Dox-regulated *Srf* followed by *ires*-*mCherry* in the *Col1a1* locus of an ESC line (carrying *M2*-*rtTA* in *Rosa26*) and injected the established cells into blastocysts to produce chimeric mice (KH2-Srf mouse; Fig. 6c and Supplementary Fig. 13a)^41^. These chimeric mice expressed the transgene in various tissues such as the epithelia of digestive tissues in response to Dox (Supplementary Fig. 13b). In the intestine, these chimeric mice reproducibly showed a colitis that resembled ulcerative colitis, a bowel disease that is caused by a poorly understood mechanism, and was characterized by signatures such as inflammatory cell infiltration, which caused dropouts of epithelia and crypt abscess in the colon (Fig. 6d and Table 3). The lesioned parts coincided well with the area where ectopic *Srf* was expressed (Fig. 6d). Hnisz et al.^42^ have reported that single-nucleotide polymorphisms (SNPs) in super-enhancers are associated with various diseases including ulcerative colitis in humans^42^. As these enhancers control cell-type-specific gene expressions, they suggested that the destabilization of cell identity can cause diseases^42^. Our results are consistent with that report and suggest that Srf misactivation is potentially involved in the pathology of ulcerative colitis by repressing cell-type-specific genes in colon tissues. Abnormalities were also reproducibly found in other tissues that expressed ectopic *Srf*, such as acinar-to-ductal metaplasia (ADM)-like alterations in the pancreas (Fig. 6d and Table 3) and dysplasia-like observations in the stomach (Supplementary Fig. 13c and Table 3), both of which are symptoms associated with tumorigenesis.

**Table 3:** Statistic analysis of phenotypes caused in *Srf*-overexpressing mice

Tissue	Dox-dosing period (days)	Phenotype	Dox +	Dox –	*P*-value
Stomach	7	Dysplasia-like	5/5	0/4	0.008
Pancreas	3	ADM-like	3/3	0/4	0.029
Colon	3	UC-like	3/3	0/4	0.029

Dox + and Dox – indicate *Srf*-overexpressing and control mice, respectively. Two-sided Fisher’s exact test. ADM, acinar-to-ductal metaplasia; UC, ulcerative colitis

To verify the possible involvement of SRF in diseases, we re-analyzed publically available human microarray data of diseases associated with SNPs in super-enhancers (see Hnisz et al.^42^) including ulcerative colitis. We performed TRANSFAC motif analyses for TF-binding sites in genes downregulated in patient tissues. In ulcerative colitis, to exclude secondary effects of inflammation, data from patients before the onset of inflammation were used^43^. Based on our analyses, we found that SRF-binding motifs were enriched in cells from various disease types, such as sigmoid colon for ulcerative colitis, T-helper cells for type 1 diabetes, hippocampus for Alzheimer’s disease, T-helper cells for multiple sclerosis, and atrial cardium for atrial fibrillation (Supplementary Fig. 14a). Moreover, cell-type-specific genes were enriched in the downregulated genes of these diseases (Supplementary Fig. 14b), consistent with Hnisz et al.^42^.

**Fig. 7:**
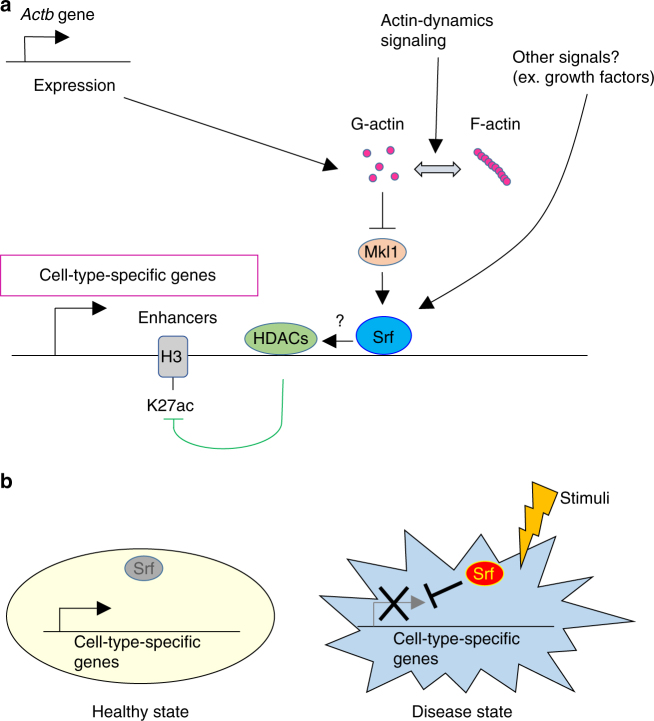
A schematic model indicating that Srf destabilizes cell identity by repressing cell-type-specific genes. **a** A model of Srf-related pathways to repress cell-type-specific gene expressions. Srf preferentially binds to open regions which preferentially contain cell-type-specific genes. Various signals, such as *Actb* expression changes and β-actin polymerization dynamics, are transduced to Srf. Srf activated by Mkl1 inactivates the surrounding regions, possibly by recruiting or activating HDACs. Other signals that regulate Srf through β-actin-independent pathways could also participate in this regulation. **b** A schematic view of a possible mechanism by which misactivation of Srf causes diseases. When the activity of Srf is at physiological levels, cell-type-specific genes are normally expressed. On the other hand, when Srf is activated at non-physiological levels (misactivation), these genes are repressed, triggering malfunction of the cells and tissues and sometimes leading to disease

As these diseases are known to be associated with SNPs in super-enhancers^42^, we next examined possible associations between Srf and super-enhancers. We used the ROSE algorithm^5,42,44^ and predicted 967 super-enhancer regions in mouse NPCs. These super-enhancers were significantly overlapped with Srf-binding sites that had been determined by ChIP-seq (Supplementary Fig. 15). Collectively, these data suggest that Srf represses cell-type-specific genes in part through cell-type-specific enhancers and/or promoters, and that appropriate Srf activity is necessary to maintain cell identity and the healthy state of an organism (Fig. 7a, b).

## Discussion

Here we demonstrate that extracellular stimuli activate Srf to suppress cell identity. We showed that the reprogramming of cells from multiple germ layers is inhibited by cell-type-specific genes, which is in agreement with a previous report^11^. We further found that a ubiquitously-expressed gene, *Actb*, which encodes β-actin, also inhibited reprogramming by regulating downstream cell-type-specific genes. In addition, the repression *of Actb* or reduction of G-actin increased the nuclear localization of Mkl1, a cofactor of Srf, to canonically activate Srf^12^. Intriguingly, we found that *Srf* overexpression alone significantly downregulated cell-type-specific genes downstream of Mkl1. Mechanistically, Srf bound to active enhancers/promoters to diminish the active mark around its binding sites and disrupt cell-type-specific patterns of the chromatin organization. Enhancers that regulate cell-type-specific genes should be vulnerable to perturbation of their components as exemplified by its binding sites and Brd4^5,42,44^. Thus, interference of Srf with any components of these enhancers could downregulate cell-type-specific genes even without changing active enhancer/promoter marks or chromatin organization. In addition, we show that the misactivation of Srf in mice caused various pathologies and found indications that Srf might contribute to the repression of cell-type-specific genes in human disease samples. These results suggest that Srf is able to suppress cell identity in a variety of cell types and that Srf activation by various stimulations in vivo can trigger various diseases.

A variety of extracellular stimuli are known to regulate Srf activity. For example, soluble factors bound to specific receptors (e.g., receptor tyrosine kinases and G-protein-coupled receptors) transduce signaling to Srf through the Rho-β-actin pathway^45^. In addition, although the expression of *Actb* is thought to be ubiquitous, the expression level changes in response to extracellular stimuli^46^, suggesting such extracellular stimuli can regulate G-actin quantity and hence Srf activity. Moreover, mechanical cues such as stiffness of the extracellular matrix and cell shape regulate Srf activity^13,40^. In this study, we show that Srf activation results in the downregulation of cell-type-specific gene expressions to promote the loss of cell identity. Taking into consideration that each cell type depends on its microenvironment, or niche^3^, this study provides a mechanism that actively destabilizes cell identity in response to changes in niche signaling (i.e., changes in soluble factors and the extracellular matrix).

Why Srf has a “dedifferentiation” function is an open question. Srf is necessary for the differentiation of various cell types, including mesoderm^47^ and epidermal cells^13^. The target genes of Srf depend on the cell type^48^ and belong to a variety of functional categories, including genes encoding AP-1 family TFs^49^, cytoskeletal proteins^33^, and developmental processes^47^. Consistently, our data showed that Srf targets cell-type-specific genes in a way that corresponds with the cell type. As promoters and enhancers of cell-type-specific genes are likely in open (active) states, Srf might preferentially (opportunistically) bind to the open and active regions of these genes (Fig. 4c), as reported for other TFs^50^.

Srf function involves the recruitment of Mkl proteins. As Mkls are relatively large molecules of around 100 kDa, their recruitment could cause steric inhibition on molecules assembled in promoters and enhancers. Alternatively, Srf may recruit repressive molecules such as HDACs. The repression of active enhancers for cell-type-specific genes promotes differentiation^51^. Thus, the physiological role of Srf may be to repress genes specific for undifferentiated cells in response to cues from the microenvironment and promote differentiation. Interestingly, MADS box family TFs, to which Srf belongs, are evolutionarily conserved among eukaryotes, from unicellular to higher metazoans, and a MADS box TF is able to promote the formation of embryonic callus (i.e., dedifferentiation) in plants^52^. Our data showed that MADS box in Srf was necessary to promote reprogramming (Fig. 3a and Supplementary Fig. 7c), suggesting that the ability of MADS box TFs to promote dedifferentiation by gene repression in response to environmental cues may be general in a wide range of eukaryotes. Further studies will be needed to clarify this mechanism.

Ulcerative colitis is a chronic autoimmune disease that sometimes causes cancer, but its original cause and pathology have remained largely unknown. This is partly due to a lack of animal models that accurately reproduce the disease. We show here that Dox-controlled *Srf*-overexpressing mice may be a useful model for ulcerative colitis (Fig. 6d and Table 3). It is possible that microenvironmental stimuli in the colon could misactivate SRF to cause a loss of cell functions, which may facilitate autoimmune responses through cell lysis^53^. Moreover, we also showed possible involvement of SRF misactivation in other diseases (Supplementary Fig. 14). A similar observation of SRF misactivation in type 2 diabetes has been reported, and SRF inhibition by a chemical compound restored the cell function (glucose uptake) in vitro and in vivo^54^. In addition, SRF activation is also suggested to be involved in the progression of fibrosis. Fibrosis is found in many diseases and increases matrix stiffness^55^. Therefore, SRF activation may exacerbate symptoms by accelerating the loss of cell functions in the affected areas. Taken together, we suggest that Srf may be a novel causative factor for a wide range of diseases, and that the Srf pathway can be an alternative target for the development of therapeutic agents.

In conclusion, we have shown that cell identity can be actively destabilized by Srf activation in response to extracellular stimuli. Multiple signals that decrease the amount of G-actin are able to activate Srf, and thus can regulate the maintenance of cell identity. Finally, proper Srf activity may be essential for the prevention of various diseases. Investigating the destabilization of cell identity in many contexts, including reprogramming, regeneration and various diseases, should reveal essential mechanisms for cell identity maintenance and advance the development of new technologies for cell reprogramming and related applications.

## Methods

### Plasmid constructions

A *piggyBac* transposon vector, PB-TA-ERN-OKMS, carrying Dox-dependent promoter-driven OKMS genes linked in tandem were constructed as follows. Using Gateway cloning with LR Clonase II (Thermo Fisher Scientific), the pENTR-OKMS entry vector^56^ was introduced into a *piggyBac* vector, PB-TA-ERN (containing a neomycin-resistant gene), which contained a constitutive rtTA expression unit and Dox-dependent expression unit^57^. For in vitro overexpression experiments using retrovirus vectors, pMXs-MCS6l-IP was constructed by replacing the neomycin-resistant gene in pMXs-MCS6l-IN^11^ to a puromycin-resistant gene. Kozak consensus sequence (CCACC) followed by open reading frames (ORFs) encoding enhanced green fluorescent protein (EGFP), β-actin, Mkl1, Elk1, Elk3, Elk4, Srf, and Srf lacking a 217 aa region from amino acid position (from the first N-terminal methionine) 2 to 218 (SrfΔN) and Srf lacking C-terminal 239 aa (SrfΔC) were inserted into the cloning site using *Sfi*I. *Actb*, *Elk1*, *Elk3*, and *Elk4* were codon-optimized for expression in mouse cells. For in vitro overexpression experiments using lentivirus vectors, pLenti6.3/V5-DEST (Thermo Fisher Scientific) was used as the backbone (destination vector). An *att*L site followed by Kozak consensus sequence, the ORFs of *egfp* and *Srf*, and another *att*L site were cloned into pUC57kan vector to produce entry vectors. The destination vector and the entry vectors were subjected to LR reaction to produce pLenti6.3/V5-DEST-EGFP and pLenti6.3/V5-DEST-Srf using LR Clonase II.

For the *Actb* and *Srf* knockdown experiments using retrovirus vectors, pSilencer 5.1-H1 Retro vector (Thermo Fisher Scientific) was used. The oligo sets shActb#1_F (5′-GAT CCG TTA AAT CTT CCG CCT TAA TAC TTC ATT CTT CCT GTC AGA AAT GAA GTA TTA AGG CGG AAG ATT TAA TTT TTA-3′)/shActb#1_R (5′-AGC TTA AAA ATT AAA TCT TCC GCC TTA ATA CTT CAT TTC TGA CAG GAA GAA TGA AGT ATT AAG GCG GAA GAT TTA ACG-3′) and shActb#2_F (5′-GAT CCG TAA TAG TCA TTC CAA GTA TCC ATG AAA TTC AAG AGA TTT CAT GGA TAC TTG GAA TGA CTA TTA TTT TTA-3′)/shActb#2_R (5′-AGC TTA AAA ATA ATA GTC ATT CCA AGT ATC CAT GAA ATC TCT TGA ATT TCA TGG ATA CTT GGA ATG ACT ATT ACG-3′) for *Actb*; shSrf#1_F (5′-GAT CCG CGG GAC TGT GCT CAA GAG TTT CAA GAG AAC TCT TGA GCA CAG TCC CGT TTT TA-3′)/shSrf#1_R (5′-AGC TTA AAA ACG GGA CTG TGC TCA AGA GTT CTC TTG AAA CTC TTG AGC ACA GTC CCG CG-3′) and shSrf#2_F (5′-GAT CCG CTG CAG CCC ATG ATC ACC ATT CAA GAG ATG GTG ATC ATG GGC TGC AGT TTT TA-3′)/shSrf#2_R (5′-AGC TTA AAA ACT GCA GCC CAT GAT CAC CAT CTC TTG AAT GGT GAT CAT GGG CTG CAG CG-3′) for *Srf*; shMkl1#1_F (5′-GAT CCG TAA CAT GTA GAC ACC TGC CAT TGC CTC TTC AAG AGA GAG GCA ATG GCA GGT GTC TAC ATG TTA TTT TTA-3′)/shMkl1#1_R (5′-AGC TTA AAA ATA ACA TGT AGA CAC CTG CCA TTG CCT CTC TCT TGA AGA GGC AAT GGC AGG TGT CTA CAT GTT ACG-3′) and shMkl1#2_F (5′-GAT CCG CAG GTA AAT TAC CCA AAG GTA TTC AAG AGA TAC CTT TGG GTA ATT TAC CTG TTT TTA-3′)/shMkl1#2_R (5′-AGC TTA AAA ACA GGT AAA TTA CCC AAA GGT ATC TCT TGA ATA CCT TTG GGT AAT TTA CCT GCG-3′) for *Mkl1*; and shEGFP_F (5′-GAT CCG ACA ACA GCC ACA ACG TCT ATA TCA TGG TCT CTT GAA CCA TGA TAT AGA CGT TGT GGC TGT TGT TTT TTA-3′)/shEGFP_R (5′-AGC TTA AAA AAC AAC AGC CAC AAC GTC TAT ATC ATG GTT CAA GAG ACC ATG ATA TAG ACG TTG TGG CTG TTG TCG-3′) and shLuc_F (5′-GAT CCG ACA ACC GCG AAA AAG TTG CTT CAA GAG AGC AAC TTT TTC GCG GTT GTT TTT TTG GAA A-3′)/shLuc_R (5′-AGC TTT TCC AAA AAA ACA ACC GCG AAA AAG TTG CTC TCT TGA AGC AAC TTT TTC GCG GTT GTC G-3′) for negative controls were annealed and ligated into the *Eco*RI/*Hin*dIII-digested plasmid to construct vectors to be used for virus production. For the knockout of *Actb* using a CRISPR/Cas9 lentivirus system, pLentiCRISPR (Addgene plasmid 49535) vector was used. The oligo sets CRISPR-Actb#1_F (5′-CAC CGG GAT GAC GAT ATC GCT GCG C-3′)/CRISPR-Actb#1_R (5′-AAA CGC GCA GCG ATA TCG TCA TCC C-3′) and CRISPR-Actb#2_F (5′-CAC CGT CGC GGG CGA CGA TGC TCC C-3′)/CRISPR-Actb#2_R (5′-AAA CGG GAG CAT CGT CGC CCG CGA C-3′) were annealed and ligated into the *Bsm*BI-digested plasmid.

### Cell culture

N31 and N31P cells are mouse NPC lines described previously by us^11^. In brief, an ESC line EB5 carrying ires-BSDpA in *Oct4* was differentiated in vitro to establish an NPC line, NSEB5-2C^11^. Dox-dependent promoter-driven OKMS genes as well as a CAG-driven rtTA transgene were conveyed by *piggyBac* transposons and separately introduced into NSEB5-2C cells by co-transfection with pCAG-PBase^11^. Cells were reprogrammed to iPSCs by the addition of Dox. N31 cells were established by the differentiation of these iPSCs. N31P cells are N31 derivatives in which the *Pax6* transgene is introduced by a *Tol2* transposon system. These cells are almost identical to N31 cells in gene expression, proliferation, and morphology, but their reprogramming efficiency is lower^11^. Therefore, N31P is better than N31 for detecting shRNAs that accelerated reprogramming. NPCs were maintained on a gelatin-coated culture plate in a NPC medium consisting of RHB basal (StemCells) containing epidermal growth factor (EGF) (Peprotech) at 10 ng/ml and human basic fibroblast growth factor (bFGF) (Peprotech) at 10 ng/ml. NPC culture also used another NPC medium, N2B27 medium, which was purchased (Takara) or prepared as a 1:1 mixture of Neurobasal (Thermo Fisher Scientific), and Dulbecco’s modified Eagle’s medium (DMEM)/F12 (1:1) with l-glutamine and sodium bicarbonate (Thermo Fisher Scientific) supplemented with 1 × N2 supplement (Thermo Fisher Scientific), 1 × B-27 supplement (Thermo Fisher Scientific), an additional 500 µM l-glutamine (Thermo Fisher Scientific), 10 µg/ml insulin (Wako), and 37.5 µg/ml bovine serum albumin (BSA) fraction V (Sigma-Aldrich) supplemented with 10 ng/ml EGF and 10 ng/ml bFGF. NSEB5-2C or N31 cells were used as NPCs for molecular biological analyses. To analyze the effect of extracellular matrix stiffness on gene expression, N31 cells and N31 cells expressing shSrf#1 (see above) were maintained in a NPC medium without EGF or bFGF on matrices (0.5, 2, or 12 kPa stiffness) lied over culture dishes coated with type I collagen (Matrigen).

Hepatoblasts HNG2 are clonally expanded primary cells from the fetal (E13.5) liver of STOCK Tg (Nanog-GFP, Puro)1Yam mice, established by us^11^. HNG2 carries the BAC-transgenic locus of Nanog-GFP, which allows us to analyze reprogramming efficiency. In HNG2, PB-TA-ERN-OKMS was introduced by lipofection with pCAG-PBase using Lipofectamine 2000 (Thermo Fisher Scientific) and 2 mg/ml G418 (Sigma-Aldrich) was added to establish HOKMSCN. These cells were maintained on a type IV collagen-coated culture plate in a medium consisting of a 1:1 mixture of DMEM/Ham’s F-12 with l-glutamine, HEPES, and sodium pyruvate (Wako) supplemented with 10% fetal bovine serum (FBS) (vol/vol) (Nichirei Bioscience), 1 µg/ml insulin (Wako), 100 nM dexamethasone (Sigma-Aldrich), 10 mM nicotinamide (Sigma-Aldrich), 50 µM β-mercaptoethanol (Nacalai Tesque), 50 ng/ml human recombinant hepatocyte growth factor (HGF; Peprotech), and 20 ng/ml EGF.

Mouse UBCs (from Kenji Osafune)^29^ were maintained on a gelatin-coated culture plate in DMEM (Wako) supplemented with 10% FBS (Thermo Fisher Scientific) and 1 mM sodium pyruvate (Nacalai Tesque). In UBCs, PB-TA-ERN-OKMS was introduced by lipofection with pCAG-PBase using Lipofectamine 2000 and 2 mg/ml G418 was added to establish UOKMSCN.

Mycoplasma contamination was checked for all the cell types using MycoAlert Mycoplasma Detection Kit (Lonza).

### Cell reprogramming

The culturing conditions for the reprogramming assays are summarized in Supplementary Fig. 16. In brief, cells were cultured in maintenance media with or without EGF, bFGF, or HGF for the first several days, then in GMEM (Wako) supplemented with 10% Knockout Serum Replacement (Thermo Fisher Scientific), 1 μM adrenocorticotropic hormone, 1 mM sodium pyruvate (Nacalai Tesque), 1 × nonessential amino acids (Nacalai Tesque), 100 μM β-mercaptoethanol, and 1000 U/ml of leukemia inhibitory factor (LIF) (Millipore), and/or N2B27 supplemented with 2i inhibitor and 1000 U/ml of LIF. The medium was replaced with ESC medium (GMEM supplemented with 10% FBS (Thermo Fisher Scientific), 1 mM sodium pyruvate, 1 × non-essential amino acids, 100 μM β-mercaptoethanol, and 1000 U/ml of LIF (Millipore)). Dox was added at the concentrations indicated in the figures. Media were replaced with fresh media every 2 or 3 days. For N31, N31P, and UOKMSCN, cultured cells were stained for alkaline phosphatase expression using ALP Staining Kit (Muto Pure Chemicals). For HOKMSCN, cultured cells were analyzed for EGFP expression as an indicator of *Nanog* expression using FACS BD Accuri (BD Biosciences). Statistical analyses were performed by F-test and one-sided Student’s *t*-test to test enhancement and repression of reprogramming efficiencies unless stated otherwise.

### Genome-wide shRNA screening

To construct *piggyBac* vectors based on a shRNA screening system, a pooled shRNA library that targets whole mouse transcripts (GeneNet Mouse 40 K, SBI) was cloned into the *Bam*HI-*Eco*RI site of the PB-H1-ccdB-EF1a-RiH *piggyBac* vector. The sequence and complexity of the cloned shRNA library was confirmed by amplicon sequencing using MiSeq. Then, the *piggyBac* shRNA library was introduced into two target cell lines, N31P cells and HOKMSCN, by the electroporation of pCAG-PBase and library plasmids using NEPA21 electroporator (Nepagene) and lipofection using Lipofectamine 2000, respectively. Cells were selected with hygromycin and cultured as summarized in Supplementary Figs. 1 and 4a, b. For NPCs, “suboptimal” concentrations of Dox (40 ng/ml) were applied to induce basal levels of OKMS. In this condition, the formation of iPSC colonies was not induced to facilitate the detection of hit shRNAs. In the screening using hepatoblasts, 1000 ng/ml Dox was applied to reprogram cells and fluorescence-activated cell sorting (FACS) was used to separate negative, low positive, middle positive, and high positive fractions for Nanog expression. Genomic DNAs were isolated using PureLink Genomic DNA Mini Kit (Thermo Fisher Scientific). To construct a library for the second round of shRNA screening with NPCs, shRNA sequences were amplified from the genomic DNA isolated from iPSC colonies in the first screening using the following primers: PB_shRNA_Fwd (5′-GGG TAG TTC TTT AGA CGA TGA GCA T-3′) and PB_shRNA_Rev (5′-GCT TGT GGT CTC TTA TAG CCG CG-3′). Amplified shRNA fragments were digested with *Bam*HI/*Eco*RI and cloned into the PB-H1-ccdB-EF1a-RiH vector. To amplify the shRNA sequences and to add the sample index for deep sequencing, the following primer sets were used: Multiplex Rd1 fwd_PB (5′-ACA CTC TTT CCC TAC ACG ACG CTC TTC CGA TCT NNN NTG TAT GAG ACC ACT TGG ATC CG-3′) (NNNN is a 4 bp sample index) and Multiplex Rd2 rev_PB (5′-GTG ACT GGA GTT CAG ACG TGT GCT CTT CCG ATC TNN NNG ATC GCC CGG GTT TGA ATT C-3′) (NNNN is an index). Next, the second PCR was performed to add adapter sequences for the Illumina sequencing reaction using the following primers: Multiplex P5 fwd (5′-AAT GAT ACG GCG ACC ACC GAG ATC TAC ACT CTT TCC CTA CAC GAC GCT C-3′) and Multiplex P7 rev (5′-CAA GCA GAA GAC GGC ATA CGA GAT GTG ACT GGA GTT CAG ACG TGT GCT C-3′). After size extraction and purification by MonoFas DNA Purification Kit I (GL Sciences), the quality and quantity of the samples were analyzed by TapeStation 2200 (Agilent Technologies) and KAPA Library Quantification Kit for Illumina (Nippon Genetics). Amplicons were subjected to deep sequencing using MiSeq (Illumina) for NPCs and HiSeq 2500 (Illumina) for hepatoblasts. Ratios of each shRNA sequence among total reads were compared between samples before and after each screening (for NPCs) or between each Nanog-positive and -negative fraction to calculate the enrichment of each shRNA sequence.

The identified genes were subjected to DAVID functional annotation analysis^18,19^ (http://david.abcc.ncifcrf.gov) and the Expression Analysis for Canonical Pathways using the IPA (Tomy Digital Biology).

### Fluorescence-activated cell sorting

Cells were suspended in FACS buffer (2% BSA in phosphate-buffered saline (PBS) (Nacalai Tesque)) and analyzed and sorted by FACS Aria II SORP (Becton, Dickinson and Company). Gating was conducted using the fluorescence intensity and positive rate of four populations: high, 18,182 mean fluorescence intensity (MFI); middle, 5985 MFI; low, 1695 MFI; and negative, 63 MFI. The positive and negative populations were separately collected using the cell sorter and used for the deep sequencing of shRNA sequences in the cells.

### Production of virus vectors

To prepare retrovirus vectors, Plat-E cells (Cell Biolabs) were seeded in DMEM medium supplemented with 10% FBS and 1 mM sodium pyruvate the day before plasmid transduction. The plasmid DNAs of pMXs- or pSilencer-based vectors were introduced into Plat-E cells using Fugene 6 transfection reagent (Promega) according to the manufacturer’s instructions. After incubation for 1 day, media were replaced with maintaining media for each cell type used. Cells were cultured for an additional 24 h and virus-containing supernatants were collected and centrifuged to remove cell contaminants. Viruses were infected with cells in medium containing 4 μg/ml polybrene (Nacalai Tesque) overnight. After replacement with fresh media, infected cells were selected by puromycin (5 µg/ml) for all analyses, except for examination of the reprogramming efficiency.

To prepare lentivirus vectors, HEK293T cells (ATCC) were seeded in DMEM supplemented with 10% FBS and 1 mM sodium pyruvate the day before plasmid transduction. Cells were co-transfected with pLenti-based vectors, psPAX2, and pCMV-VSV-G vector DNAs using Lipofectamine 2000. After incubation for 1 day, the medium was replaced with NPC medium without EGF or bFGF for NPCs, hepatoblast medium without EGF or HGF for hepatoblasts, or DMEM supplemented with 10% FBS for kidney cells. Cells were cultured for an additional 24 h and virus-containing supernatants were collected and centrifuged to remove cell contaminants. These virus solutions were dispensed into aliquots and kept at – 150 °C until use. Cells were infected with viruses overnight.

### Reverse transcription-quantitative PCR

Total RNAs were isolated using RNeasy Mini Kit (Qiagen) according to the manufacturer’s recommendations. First-strand DNAs were synthesized by reverse transcription using SuperScript III reverse transcriptase (Thermo Fisher Scientific). A mixture of oligo(dT)_20_ and random hexamer was used as the primers (Thermo Fisher Scientific). Using these templates, quantitative PCR (qPCR) was performed using GoTaq qPCR Master Mix (Promega) in StepOne Real-time PCR System (Thermo Fisher Scientific). The primer sets used were Gapdh_F (5′-GTG TTC CTA CCC CCA ATG TGT-3′) and Gapdh_R (5′-ATT GTC ATA CCA GGA AAT GAG CTT-3′) for *Gapdh*; Actb_F (5′-AGC TGT GCT ATG TTG CTC TAG ACT T-3′) and Actb_R (5′-CAT AGA GGT CTT TAC GGA TGT CAA C-3′) for *Actb*; Srf_in3UTR_F (5′-TTC CCG TCC GAG GAA ACA T-3′) and Srf_in3UTR_R (5′-GGC TCT TTT GAC CCA GAC CAT-3′) for *Srf*; Acta2_F (5′-GTG CTA TGT AGC TCT GGA CTT TGA-3′) and Acta2_R (5′-TAG CAT AGA GAT CCT TCC TGA TGT C-3′) for *Acta2*; Myl9_F (5′-CTC AGG CTT CAT CCA CGA G-3′) and Myl9_R (5′-GTA GTT GAA GTT GCC CTT CTT ATC A-3′) for *Myl9*; Tagln_F (5′-ACT AGT GGA GTG GAT TGT AGT GCA G-3′) and Tagln_R (5′-TCC TTA CCT TCA TAG AGG TCA ACA G-3′) for *Tagln*; and Cnn1_F (5′-AAG GTC AAT GAG TCA ACT CAG AAC T-3′) and Cnn1_R (5′-AGG AGA GTG GAC TGA ACT TGT GTA T-3′) for *Cnn1*.

### Western blot analyses

For the western blotting analysis, protein samples were prepared by lysing cells with RIPA buffer (20 mM Tris-HCl, 100 mM NaCl, 0.1% (w/v) SDS, and 1% (v/v) Nonidet P-40 (NP-40) pH 8.0), except for the detection of H3K27ac, in which case nucleic lysis buffer (10 mM Tris-HCl, 10 mM EDTA, 1% SDS, and 200 mM NaCl pH 7.5) was used. Cell contaminants were removed by centrifugation. The samples and the size marker (Bio-Rad, 161-0376) were resolved on 4–20% gradient SDS-polyacrylamide gel (Mini-PROTEAN TGX; Bio-Rad). Proteins were transferred to a polyvinylidene difluoride membrane (Trans-Blot Turb Transfer Pack; Bio-Rad), which was subsequently blocked in Blocking One (Nacalai Tesque) and incubated overnight at 4 °C with primary antibodies anti-β-actin (produced in mouse; AC-74; Sigma-Aldrich, A5316), anti-Srf (produced in rabbit using residues surrounding Ser375 of human SRF as an immunogen; D71A9; Cell Signaling Technology, 5147), anti-Srf (produced in rabbit using a.a.65-95 of human SRF as an immunogen; Sigma-Aldrich, SAB4502852), anti-H3K27ac (produced in rabbit; D5E; Cell Signaling Technology, 8173), and anti-Gapdh (produced in mouse; 6C5; Millipore, CB1001) at a 1:1000 dilution. After washing three times with TBST (1 × TBS (Bio-Rad) containing 0.1% Tween 20), the membrane was incubated with a secondary antibody for the size marker (Bio-Rad, 161-0380) and an horseradish peroxidase (HRP)-conjugated goat anti-rabbit or mouse IgG secondary antibody (Santa Cruz Biotechnology, sc-2004 and sc-2005, respectively) at a 1:000 dilution at room temperature for 1–3 h and washed again. A chemiluminescent reagent (Clarify Western ECL Substrate; Bio-Rad) was subsequently applied onto the blotted membrane. The luminescence signal was detected using a chilled charge-coupled device digital imaging camera (LAS4000; Fujifilm). Full blot images are shown in Supplementary Fig. 17. For H3K27ac and Gapdh, bands were quantified using Image J. Statistical analyses of the band intensities of H3K27ac per those of Gapdh were performed by *F*-test and two-sided Student’s *t*-test.

### Immunocytochemistry

Each of control, *Actb*-knockout and *Srf*-overexpressing NPCs were seeded on a gelatin-coated culture plate. Cells were fixed with 4% paraformaldehyde (Nacalai Tesque) and permeabilized with 0.5% TritonX-100 in PBS. These cells were treated with Blocking One (Nacalai Tesque) and reacted with Rabbit anti-Mkl1 antibody (Abcam, 49311) at a 1:200 dilution overnight. Specimens were subsequently reacted with Alexa Fluor 594-conjugated goat anti-rabbit IgG (Thermo Fisher Scientific, A-11012) at a 1:500 dilution and mounted with VECTASHIELD Mounting Medium (Vector Laboratories) followed by observation using an inverted IX73 fluorescence microscope (Olympus).

### Microarray

Total RNA was extracted using RNeasy Mini Kit and assessed using RNA 6000 LabChip kit and Agilent 2100 Bioanalyzer (Agilent Technologies). High-quality RNA was used in the subsequent experiments. Microarray experiments used systems of two manufacturers (Agilent Technologies and Affymetrix) and were performed as follows. For Agilent Technologies products, 200 ng of total RNA was reverse-transcribed and amplified using Low RNA Input Fluorescent Linear Amplification Kit. The resultant cDNA was labeled with Cyanine 3-CTP, hybridized to SurePrint G3 Mouse GE 8 × 60 K, and washed following the manufacturer’s instructions. The cDNA were scanned with an Agilent Scanner. Fluorescence intensities of the scanned images were subsequently quantified using Feature Extraction software. For Affymetrix products, 200 ng of total RNA was subjected to cDNA synthesis with GeneChip WT PLUS Reagent Kit and the resultant cDNA was fragmented and hybridized to Mouse Gene 2.0 ST Array. After hybridization, GeneChip arrays were washed and stained by GeneChip Fluidics Station 450 and detected by Scanner 3000 TG system following the manufacturer’s standard protocols. The array data were analyzed for comparison among samples using GeneSpring software. Gene expression values were normalized by the exclusion of low-signal-intensity data and percentile shifts. The tissue expression of the genes was analyzed using DAVID functional annotation analysis^18,19^.

### Chromatin immunoprecipiation sequencing

ChIP-seq analysis was performed according to a previous report with minor modifications^11^. Around 5.0 × 10^6^ cells per 10 cm dish were fixed in 1% paraformaldehyde at room temperature for 10 min followed by quenching with 125 mM glycine at room temperature for 5 min. Fixed cells were washed with cold PBS twice. Cells were collected by scraper and centrifuged at 700 × *g* for 3 min and then resuspended in nucleic lysis buffer (10 mM Tris-HCl, 10 mM EDTA, 1% SDS and 200 mM NaCl pH 7.5). The genomic DNA was fragmented with Bioruptor (Cosmo Bio) at 4 °C to a size range between 300 and 600 bp. Cell contaminants were removed by centrifugation. A part of the cleared solution was used as the input sample. An anti-SRF antibody (clone D71A9; Cell Signaling Technology, 5147) at a 1:20 dilution or anti-Histone H3 acetyl Lys27 antibody (Active Motif, 39133) at a 1:50 dilution were prebound by incubating with Protein-G Dynabeads (Thermo Fisher Scientific) in immunoprecipitation (IP) dilution buffer (16.7 mM Tris-HCl, 1.2 mM EDTA, 1.1% (vol/vol) Triton X-100, 167 mM NaCl, and 0.01% SDS pH 8.0) and then incubated at 4 °C overnight with constant rotation. Samples were washed twice with IP dilution buffer, twice with low NaCl buffer (20 mM Tris-HCl, 2 mM EDTA, 1% Trion X-100, 150 mM NaCl and 0.1% SDS pH 8.0), twice with high NaCl buffer (20 mM Tris-HCl, 2 mM EDTA, 1% Trion X-100, 500 mM NaCl and 0.1% SDS pH 8.0), twice with LiCl buffer (10 mM Tris-HCl, 1 mM EDTA, 250 mM LiCl, 1% NP-40 and 1% sodium deoxycholate pH 8.0), and twice with TE (10 mM Tris-HCl and 1 mM EDTA pH 8.0). The DNA–protein complex was finally eluted with elution buffer (25 mM Tris-HCl, 5 mM EDTA and 0.5% SDS pH 7.5). Eluates and input samples were treated with Pronase (Roche) at 42 °C for 2 h followed by 65 °C overnight with constant shaking. DNA was purified with MinElute Reaction Cleanup Kit (Qiagen). Immunoprecipitated DNA was ligated with adaptors of Illumina TruSeq using TruSeq ChIP Sample Prep Kit (Illumina). Libraries were assessed for quality and quantity using Agilent Bioanalyzer 2000 and KAPA Library Quantification Kit for Illumina (KAPA Biosystems). The libraries were single-end (50 bp) sequenced on NextSeq 500 or Illumina HiSeq 2500.

### ChIP-seq data analysis

All the sequenced reads were surveyed and the low-quality bases at the 3′-read ends and the adaptors were trimmed using cutadapt-1.14^58^. Trimmed reads with < 20 bp were discarded. Untrimmed and trimmed reads of 20 bp or longer were mapped to mouse genome (mm9) with BWA 0.7.12^59^ using the default parameters. The reads with high mapping quality (MAPQ > = 20) were used for further analysis and the duplicate reads were removed with Picard tools (http://broadinstitute.github.io/picard/). MACS ver. 1.3.7.1^60^ with parameters (--mfold = 8 --gsize = 2.43e+09 --pvalue 1e-5) was used to identify enriched regions over input samples. Peaks falling within blacklisted regions (https://sites.google.com/site/anshulkundaje/projects/blacklists) were excluded from further analysis. Binding sites were defined as sites detected in two independent experiments. Average profiles and heatmaps for ChIP-seq data were generated by ngsplot-2.47^61^. The mergePeaks program (HOMER software v4.9.1^62^) was used to test the statistical significance of the overlap. Super-enhancers were identified by H3K27ac with ROSE pipelines^5,42,44^.

### Motif analysis

Promoter sequences (from – 1000 to + 200 bp relative to TSS) were obtained by using data from the RefSeq database (https://www.ncbi.nlm.nih.gov/refseq/). The nucleotide sequences (from – 25 to + 25 bp or from – 100 to + 100 bp relative to the peak center of the ChIP-seq data) were obtained for co-enrichment analysis of the TFs. To identify putative binding sites of DNA-binding proteins, the sequences were scanned with MATCH programs^63^ (cut-off = minFP) using vertebrate position weight matrices from the TRANSFAC Professional database (BioBase)^64^. For each matrix, the enrichment of the hit sequences in a set of sequences was compared with that in whole genomic regions and *P*-values were calculated by Fisher’s exact test.

### Chromatin accessibility assay

Five thousand cells were isolated from each sample and lysed in 5 to 10 μl of ice-cold ATAC lysis buffer (10 mM Tris-HCl, 10 mM NaCl, 3 mM MgCl_2_, and 0.1% IGEPAL CA-630 pH 7.5) for 10 min. The nuclear extraction step was skipped and Illumina’s adaptors were ligated using the transposase reaction mix of Nextera DNA Sample Prep Kit (Illumina), and the samples were incubated for 30 min at 37 °C. Indices were incorporated using Nextera Index Kit (Illumina) in the amplification step. The libraries were purified using Ampure XP beads (Beckman Coulter) to remove remaining adapters. The libraries were paired-end sequenced on Illumina HiSeq 2500 for 64 cycles. Sequenced reads were handled as described in ChIP-seq data analysis above, except that the reads mapping to the mitochondrial DNA (chrM) were excluded for further analysis. ATAC-seq peak sites were defined as sites detected in two independent experiments.

### Hi-C experiments

Cells were fixed with 1% formaldehyde for 10 min at room temperature. Glycine was added to 125 mM final concentration and incubated for 5 min at room temperature. Tubes were placed on ice for 15 min. Cells were washed in ice-cold PBS and 1–2 × 10^6^ fixed cell pellets were used for the following steps. Cell pellets were resuspended in 3 C lysis buffer (10 mM Tris-HCl, 10 mM NaCl, and 0.2% NP-40 pH 8.0) with 1 × protease inhibitor cocktail (Roche) and incubated on ice for 20 min. After centrifugation, pellets were resuspended in 1.2 × NE Buffer 2 (New England Biolabs) with 0.3% SDS and incubated for 1 h at 37 °C with constant shaking. Triton X-100 was subsequently added to a 2% final concentration, and samples were incubated for 1 h at 37 °C. Samples were further incubated overnight at 37 °C with shaking after the addition of *Hin*dIII. After centrifugation, pellets were sequentially incubated in 1 × NE Buffer 2 with 300 U *Hin*dIII for over 8 h at 37 °C with constant shaking in “Fill-in Mix” (1 × NE Buffer 2, 15 µM each of dATP, dGTP, dTTP, and 15 µM Biotin-14-dCTP (Thermo Fisher Scientific) with 25 U Klenow (New England Biolabs)) for 45 min at 37 °C and in “Ligation Mix” (1 × T4 DNA ligase buffer (New England Biolabs) with 60 U/l of T4 DNA ligase (Thermo Fisher Scientific)) overnight at 16 °C. After Tris-HCl (pH 7.5) was added at 10 mM, samples were treated with 100 µg/ml RNase A for 30 min at 37 °C, subjected to reverse-crosslinking and proteinase K treatment overnight at 65 °C in the presence of 400 µg/ml proteinase K followed by incubation in 800 µg/ml proteinase K for an additional 2 h. DNA was extracted by ethanol precipitation after treatment with phenol/chloroform twice and with chloroform once, and resolved in 10 mM Tris-HCl (pH 7.5) (Hi-C DNA). Hi-C DNAs were checked for quantity and quality by Qubit dsDNA HS Assay Kits (Thermo Fisher Scientific) and electrophoresis, respectively.

To generate next-generation sequencing libraries, Hi-C DNAs were mixed with “T4 DNA pol mix” (1 × NE Buffer 2, 1 × BSA, 100 µM dATP, 100 µM dGTP, and 2.5 U of T4 DNA polymerase (New England Biolabs)) and incubated for 2 h at 12 °C. These DNAs were purified by ethanol precipitation after treatment with phenol/chloroform twice and with chloroform once, and resolved in nuclease-free water. DNAs were next sheared (300–500 bp) using the DNA Shearing System S220 (Covaris) and subjected to size selection using AMPure XP beads (Beckman Coulter). The size-selected DNAs were subjected to biotin pull-down using Dynabeads MyOne Streptavidin C1 (Thermo Fisher Scientific). The sequencing libraries were prepared using Nextera Mate Pair Sample Prep Kit (Illumina) and KAPA Real-Time Library Amplification Kit (KAPA Biosystems), and purification was done with AMPure XP beads. The libraries were checked for quality using a Bioanalyzer High Sensitivity DNA chip and subjected to paired-end sequencing (100-bp read length) using HiSeq 2500.

### Hi-C data analysis

Read pairs were individually mapped to the mouse genome (UCSC mm9) using the Hi-C-pro pipeline^65^ as the default parameter of bowtie2 options (Global options: “--very-sensitive -L 30 --score-min L,-0.6,-0.2 --end-to-end”; Local options: “--very-sensitive -L 20 --score-min L,-0.6,-0.2 --end-to-end”). After read mapping, each side of the mapped reads was applied to the hiclib pipeline^66^. First, uniquely mapped paired-reads were assigned to *Hin*dIII fragments. Only those with correct orientations were considered valid pairs, and non-ligated and self-ligated read pairs were filtered out. Fragment-level filters were next applied to remove invalid reads: reads that started within 5 bp from the restriction sites, duplicated read pairs, extremely large and small restriction fragments ( > 100 kbp and < 100 bp, respectively), and extremely high and low count restriction fragments (top 0.5% of all counts and zero counts, respectively). Biological replicates were merged, and Hi-C contact heatmaps were generated in 200 kb non-overlapping genomic bins for both merged samples (mainly used for visualization) and for single biological replicates. To correct the bias of the contact heatmaps, iterative correction was performed with the following steps: diagonal removal was applied to only the first diagonal (“removeDiagonal(0)”), bin removal was done by the sequence fraction (0.5), and finally high trans contacts (the fraction of the top 0.05% trans contacts) were removed. The bias-corrected contact heatmaps were used to generate the A/B compartment profiles in each chromosome as previously described^37^ by the hiclib pipeline with a small modification in the ∆“doCisPCADomains(domaidunction = ”lieverman-”)” function including observed/expected analysis, covariance matrix generation from the Pearson’s correlation matrix, and PCA analysis. The first eigenvalues were used to identify A/B compartments. As positive and negative eigenvalues are arbitrary, correlation with the GC content was used to assign positive eigenvalues as ‘A compartment’ and negative eigenvalues as ‘B compartment’.

Genomic regions with significant A/B compartment changes in 200 kb bins were identified as previously described^38^. First, one-way analysis of variance (ANOVA) test (‘oneway.test’ function in R) was performed for each 200 kb genomic bin among control (NPCs), *Actb* knockout (Actb KO), and *Srf* overexpression (Srf OE) sample sets to identify genomic regions that exhibit statistically significant variability in A/B compartments (*P* < 0.05). Second, genomic regions with either A-to-B or B-to-A compartment changes were selected by comparing the NPCs with Actb KO or Srf OE in both biological replicates. The Refseq gene positions (UCSC mm9) were assigned to each 200 kb genomic bin.

For Hi-C data analysis of compartment changes during the reprogramming to iPSCs, the same analysis as above was performed, and A/B compartment profiles were generated by comparing our NPC data to NSC-derived iPSC data^39^, NSC_iPS_p3 (GEO accession numbers: GSM2026282, GSM2026283, GSM2026284) and NSC_iPS_p20 (GSM2026279, GSM2026280, and GSM2026281). Specifically, the summary mapped txt files (GSE76481)^39^ were loaded to the hiclib pipeline using our home-made python script. For the NSC-derived iPSCs data sets, *Dpn*II was used as the restriction sites. In ANOVA tests, the two parental NPCs, NSC_iPS_p3, and NSC_iPS_p20 data sets were used. For the creation of figures, the merged data sets were used for visualization.

To estimate the association of compartment changes with cell-type-specific genes, the number of genes was counted after excluding redundant genes, genes that were not found in the microarray data and genes annotated with no gene symbol.

### RNA sequencing

RNA sequencing (RNA-seq) was performed to analyze gene expressions in cells on matrices of different stiffness. One hundred nanograms of total RNA isolated using RNeasy Mini Kit was used and the libraries were constructed using TruSeq Stranded mRNA LT Sample Prep Kit (Illumina). Libraries were assessed for quality and quantity using Agilent Bioanalyzer 2100 and Qubit dsDNA HS Assay Kit (Thermo Fisher Scientific). The libraries were single-end sequenced on Illumina NextSeq 500. The sequenced reads were mapped to the mouse reference genome (mm10) using tophat-2.1.0^67^ with the GENCODE version M14 annotation gtf file and the aligner Bowtie2-2.2.8^68^ after trimming adaptor sequences and low-quality bases by cutadapt-1.9.1^58^. The reads with high mapping quality (MAPQ ≥ 20) were used for further analyses. The expression level of each gene was calculated as reads per kilobase per million mapped reads (RPKM) by cufflinks-2.2.1^69^ and analyzed using GeneSpring software. Genes whose RPKM was > 2 in all samples were included. Genes expressed in control NPCs on the 0.5-kPa matrix more than 2-fold compared with ESCs (GSM1891554 and GSM1847711) were defined as cell-type-specific genes on the 0.5 kPa matrix (these genes are not the same as the cell-type-specific genes on normal culture dishes that were used for the experiments, except for experiments that examined the stiffness effects on cell identity, because the gene expression programs were different). The threshold was 1.1-fold for the selection of downregulated and upregulated genes.

### ESC targeting and generation of chimeric mice

To generate Srf-inducible mice, the Dox-inducible system was employed (Fig. 6c). The *Srf*-ires-*mCherry*-pA sequence was inserted into pBS31^41^, which was electroporated into KH2 ESCs (mixed background of 129 and BL/6 strains, from Konrad Hochedlinger) harboring a *Rosa26*-promoter-driven *M2rtTA* allele as described previously^41^. Established KH2-Srf ESCs (*Rosa*-*M2rtTA*/*Col1a1*::tetO-*Srf*-ires-*mCherry*) were injected into ICR mouse blastocysts to obtain chimeric mice, in which Srf can be induced in vivo by adding Dox in drinking water. Four-week-old animals with similar chimerism were used for the analyses. From them, randomly (without blinding) selected mice were treated with Dox at 2 µg/ml for 4 or 7 days before subjected to observations for Srf overexpression (mCherry expression) and phenotypic diagnosis. All experimental groups contained both females and males.

To determine the differentiation capability of shActb-iPSCs in vivo, cells were first labeled with EGFP by introducing a *Tol2* transposon vector carrying CAG-*egfp*-ires-*pac* (puromycin resistance gene) by co-transfection with pCAGGS-mT2TP^11,70^ using Lipofectamine 2000. EGFP-labeled shActb-iPSCs were injected into blastocysts to obtain E14.5 chimeric embryos. The chimeric contribution of shActb-ESCs was analyzed by immunohistochemistry using an anti-GFP antibody (see below) (clone 4B10; Cell Signaling Technology).

All experiments using animals were conducted under the ethical guidelines of Kyoto University.

### Histological analysis and immunohistochemistry

All tissue samples were fixed in 4% paraformaldehyde overnight and embedded in paraffin. Sections (5 μm) were stained with hematoxylin and eosin using standard protocol, and serial sections were used for immunohistochemical staining. The primary antibodies, which were incubated at room temperature for 1–2 h in blocking buffer (2% BSA in PBS), were rat anti-Red Fluorescent Proteins (clone 5F8, dilution 1:100, Chromotek, 5F8) or mouse anti-GFP (clone 4B10, dilution 1:100, Cell Signaling Technology, 2955). The sections were subsequently incubated with HRP-conjugated secondary antibodies (Histofine Simple Stain; Nichirei Bioscience) at room temperature for 30 min, followed by chromogen development using DAB (Nichirei Bioscience). The stained sections were counterstained with Meyer hematoxylin. Specimens were observed with a reverse BX51 microscope (Olympus).

### Data availability

Microarray, ChIP-seq, ATAC-seq, RNA-seq, and Hi-C data have been deposited in NCBI Gene Expression Omnibus under GSE90034. Other data that support the findings of this study are available from the corresponding authors upon reasonable request.

## Electronic supplementary material

Supplementary Information(PDF 1157 kb)

Description of Additional Supplementary Files(PDF 4 kb)

Supplementary Data 1(XLSX 99 kb)

Supplementary Data 2(XLSX 37 kb)

Supplementary Data 3(XLSX 64 kb)
