# Supplementary material for: Srf destabilizes cellular identity by suppressing cell-type-specific gene expression programs
Source: Nat Commun. 2018 Apr 11;9:1387. doi: 10.1038/s41467-018-03748-1 (PMC5895821; doi:10.1038/s41467-018-03748-1)
Supplement: Supplementary file 1 — Supplementary Information(PDF 1157 kb) [file 41467_2018_3748_MOESM1_ESM.pdf]

SUPPLEMENTARY INFORMATION

Srf destabilizes cellular identity by suppressing cell-type-specific gene expression programs

Ikeda *et al*.

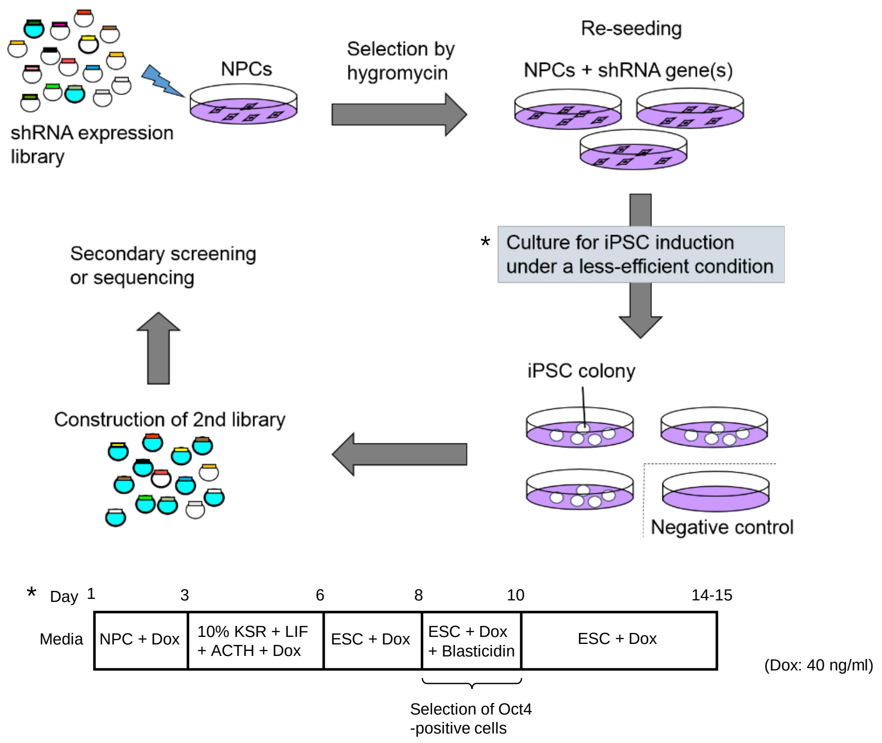

Supplementary Figure 1. Schematic view of the shRNA screenings of NPCs.

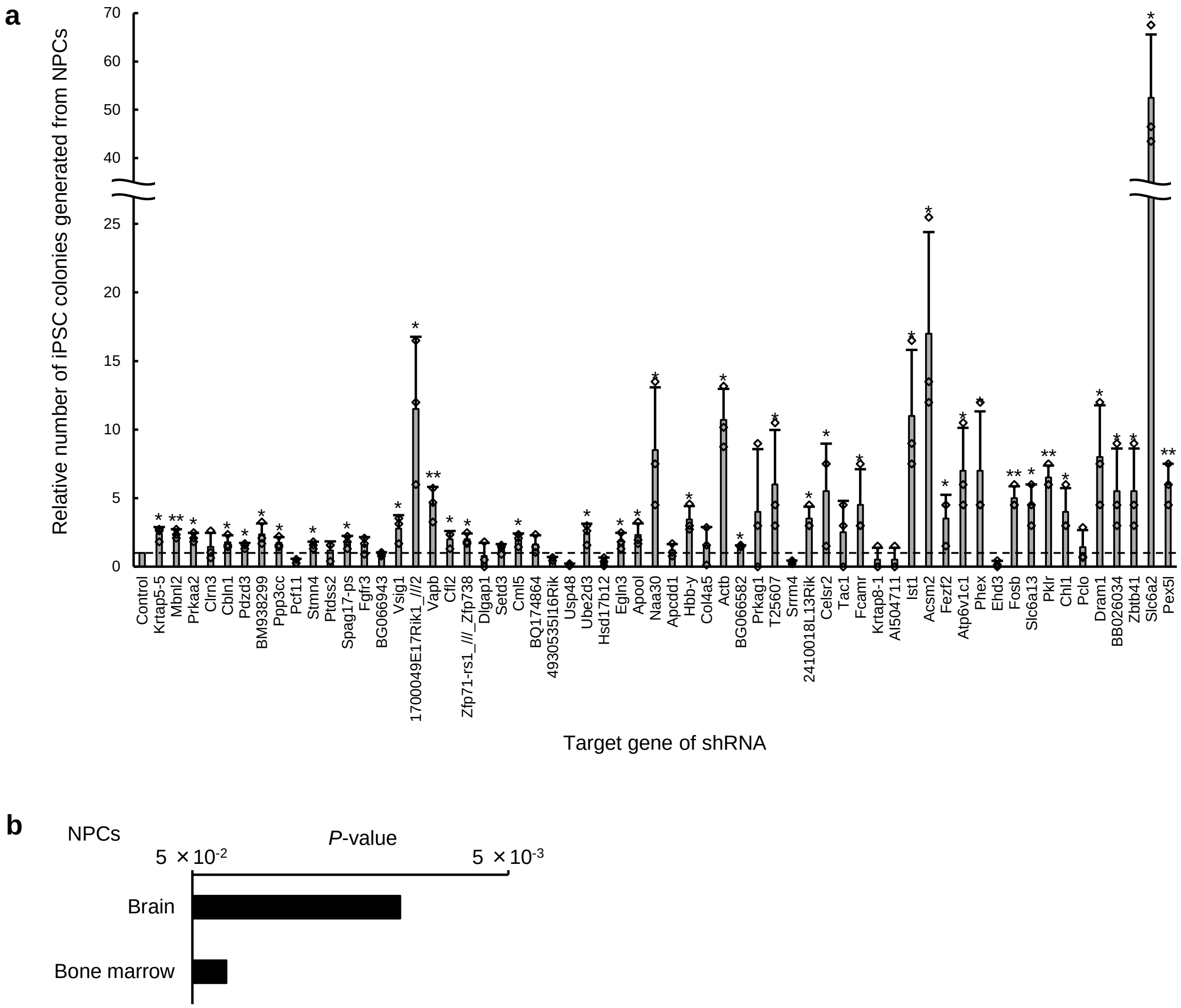

Supplementary Figure 2. Validation of the shRNA screening of NPCs. (**a**) Identified shRNAs from the 2nd screening were individually introduced into NPCs and validated for reprogramming enhancement. Values are means ± s.d. of numbers of iPSC colonies relative to the control (n=3). Dots indicate individual data points. Student's *t*-test (***P* < 0.005, **P* < 0.05). (**b**) Tissue expression analysis[1,2] of genes whose shRNA showed statistically significant reprogramming enhancement in a.

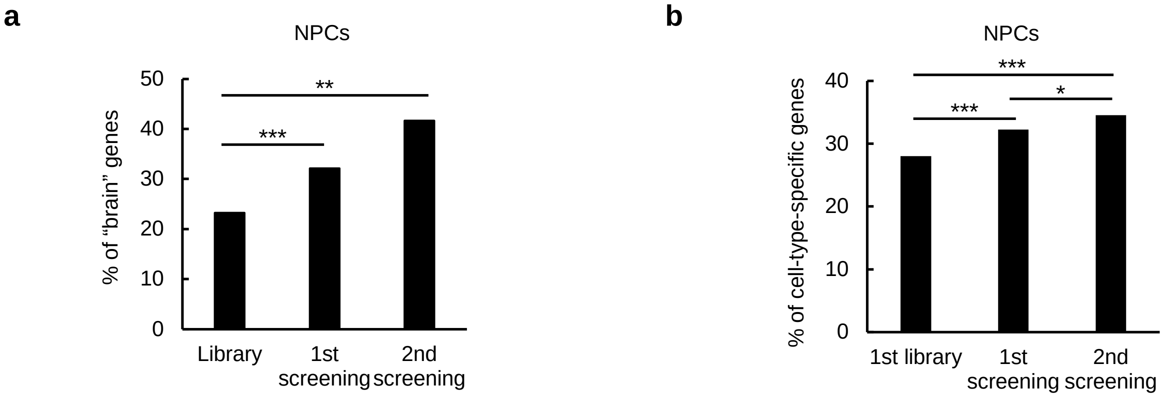

Supplementary Figure 3. Cell-type-specific genes are enriched by sequential screenings of NPCs. (**a**) "Brain" genes were enriched by sequential screenings of NPCs. The percentages of genes annotated with "brain" by tissue expression analyses[1,2] for the gene library before screening, genes from the 1st screening and genes from the 2nd screening. Two-sided Fisher's exact test (***P < 0.0005, **P < 0.005). (**b**) NPC genes were enriched by the sequential screenings of NPCs. The percentages of cell-type-specific genes in the gene library before screening, genes from the 1st screening and genes from the 2nd screening. Genes expressed in NPCs more than 2-fold compared with ESCs are defined as cell-type-specific genes. Two-sided Fisher's exact test (***P < 0.0005, **P < 0.005, *P < 0.05).

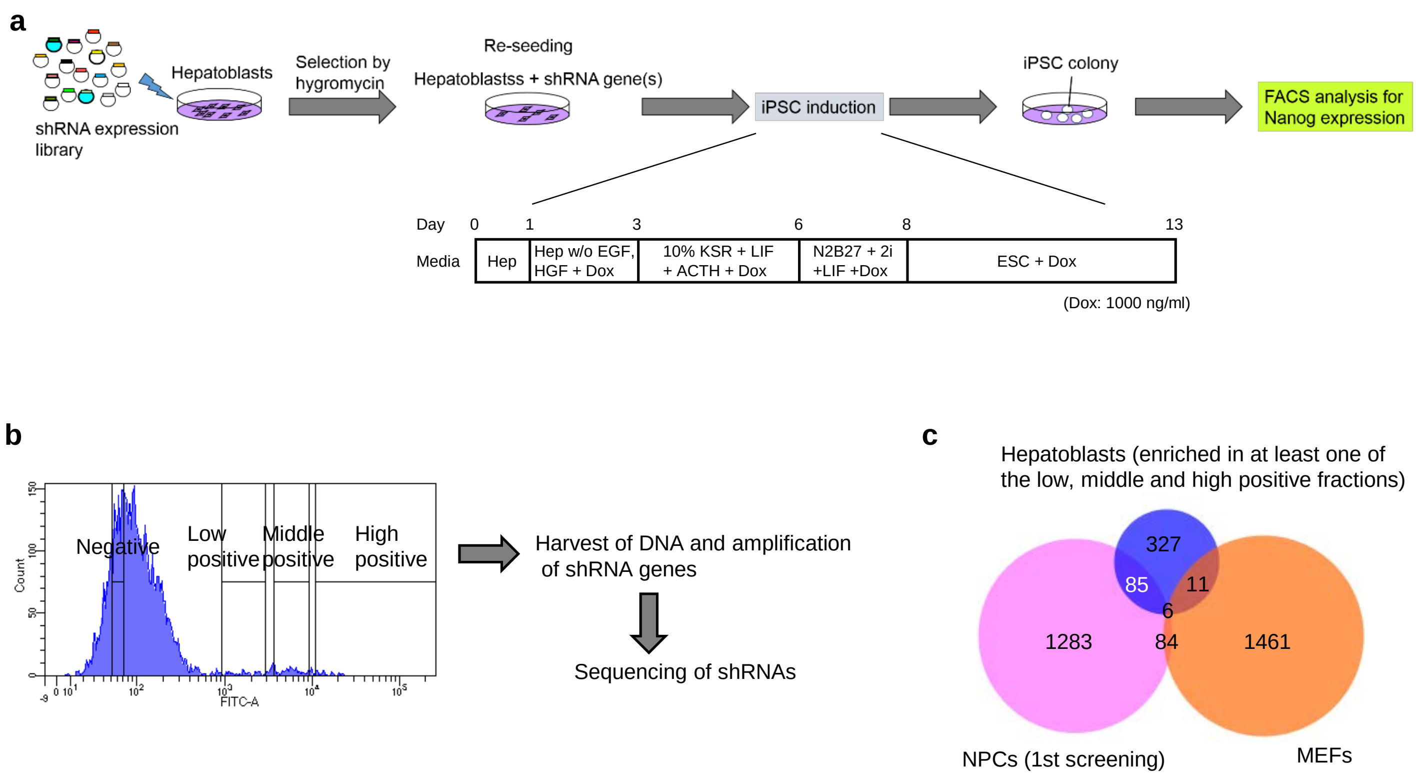

Supplementary Figure 4. Additional information on the shRNA screenings of hepatoblasts and NPCs for the identification of factors that inhibit cell reprogramming. (**a**) Schematic view of cell culture in the shRNA screenings of hepatoblasts. (**b**) Schematic view for the acquisition of shRNA sequences. The results of FACS of Nanog-positive cells generated from shRNA-library-introduced hepatoblasts after culture for reprogramming and the steps after sorting. (**c**) Factors that inhibit cell reprogramming are different among cell types even by screenings at low stringency. Genes identified from the 1st screening of NPCs, genes enriched in at least one of the positive fractions in hepatoblasts and genes enriched in the screening of MEFs reported by Yang *et al*. (2014)[3] were used to generate the Venn diagram. Numbers indicate number of genes.

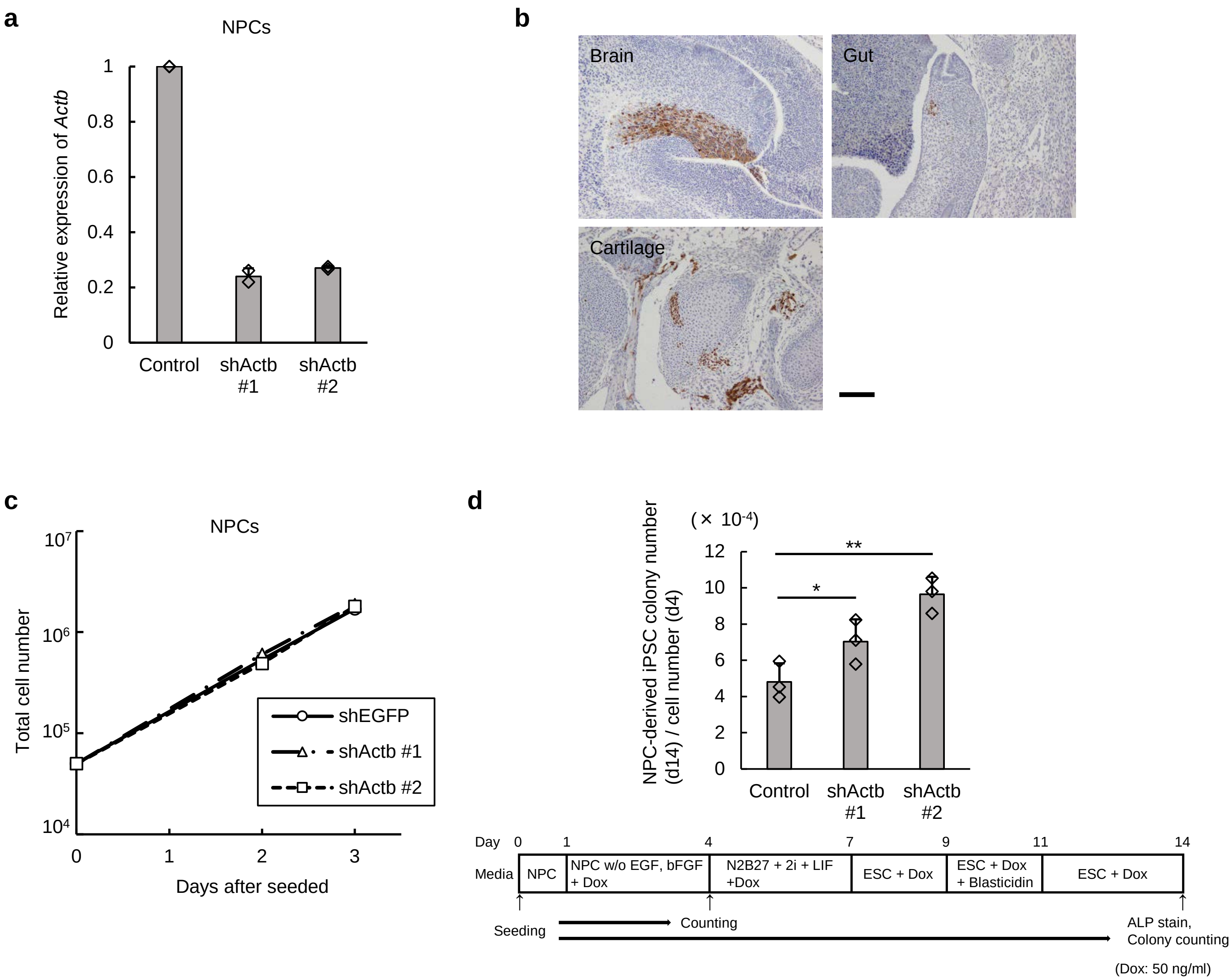

Supplementary Figure 5. Additional information on *Actb* repression in NPCs. (**a**) shActb sequences effectively repressed *Actb*. The expression level of *Actb* mRNA in NPCs with two different sequences of shRNAs for *Actb* (shActb) were analyzed by RT-qPCR. Values are means ± s.d. of expression levels of *Actb* normalized to *Gapdh* expression in NPCs relative to the ratio in control cells (n=2, technical duplicate). Dots indicate individual data points. (**b**) Reprogrammed cells established in the presence of shActb are pluripotent. Immunohistochemistry of chimeric mice produced using cells reprogrammed from NPCs expressing shActb and *egfp* was performed using an anti-EGFP antibody. Bar, 100 μm. (**c**) Knockdown of *Actb* in NPCs has little influence on cell proliferation. Values indicate means ± s.d. (n=3). Vertical axis is log scale. (**d**) The enhanced efficiency of reprogramming from NPCs is independent of proliferation. Values in the graph are means ± s.d. of the iPSC colony number at day 14 normalized to the total cell number at day 4 averaged from three samples (n=3). Dots indicate individual data points. Vertical axis is log scale. Student's *t*-test (***P* < 0.005, **P* < 0.05).

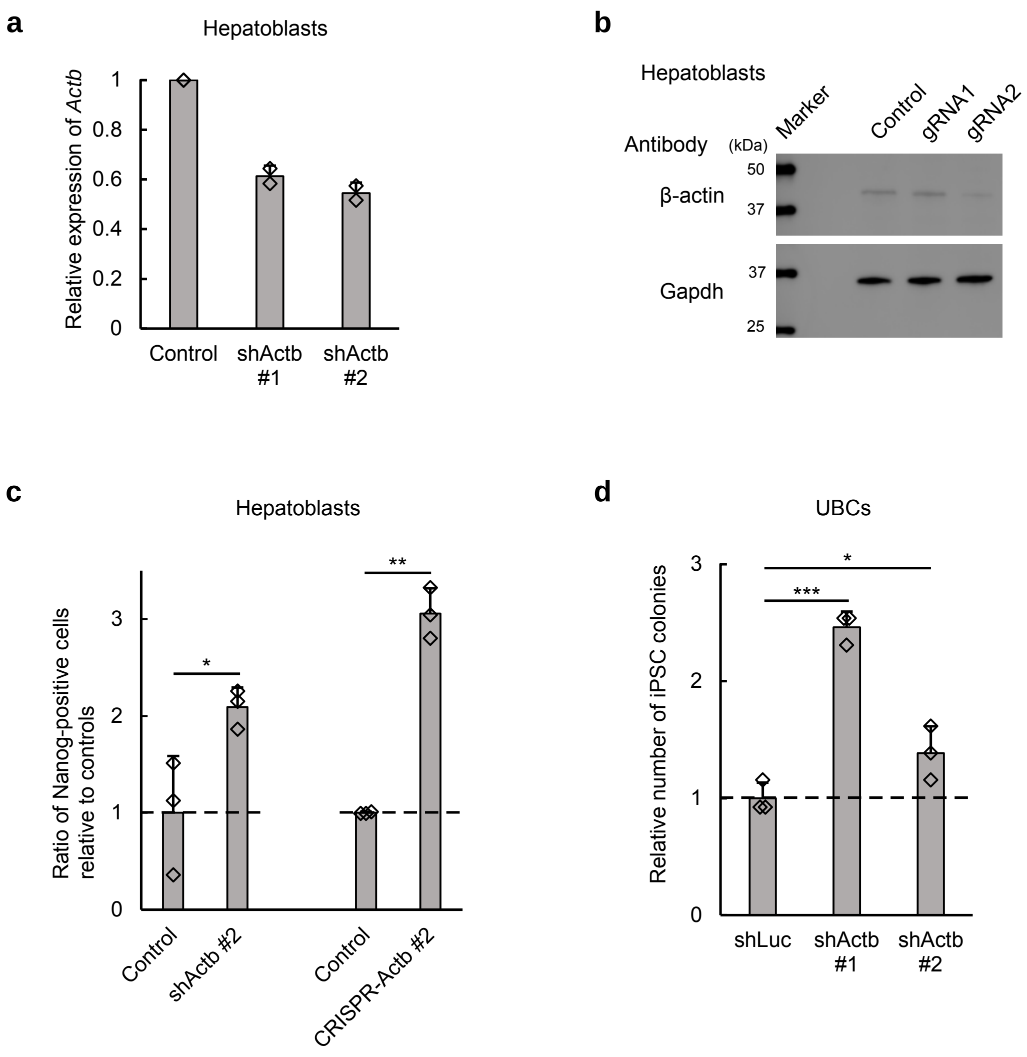

Supplementary Figure 6. Additional information on *Actb* repression in hepatoblasts and UBCs. (**a**) Repression of *Actb* in hepatoblasts. The two shActb downregulate *Actb* less efficiently in hepatoblasts. The expression level of *Actb* mRNA in cells with two different sequences of shActb were analyzed by RT-qPCR. Values are means ± s.d. of *Actb* expression levels normalized to *Gapdh* expression in shActb-introduced hepatoblasts relative to the ratio in control cells (n=2, technical duplicate). (**b**) A CRISPR/Cas9 system targeting *Actb* gene represses *Actb* expression. Immunoblotting shows that the use of gRNA2 effectively represses β-actin expression in hepatoblasts. (**c**) Repression of *Actb* enhances cell reprogramming of hepatoblasts. Values are means ± s.d. of the ratio of *Nanog*-positive cells relative to controls. (**d**) Repression of *Actb* enhances cell reprogramming of UBCs (kidney cells). Values are means ± s.d. of the ratio of iPSC colonies relative to controls. Dots indicate individual data points. Asterisks indicate statistically significant differences compared to controls (Student's *t*-test; $***P < 0.0005$, $**P < 0.005$, $*P < 0.05$).

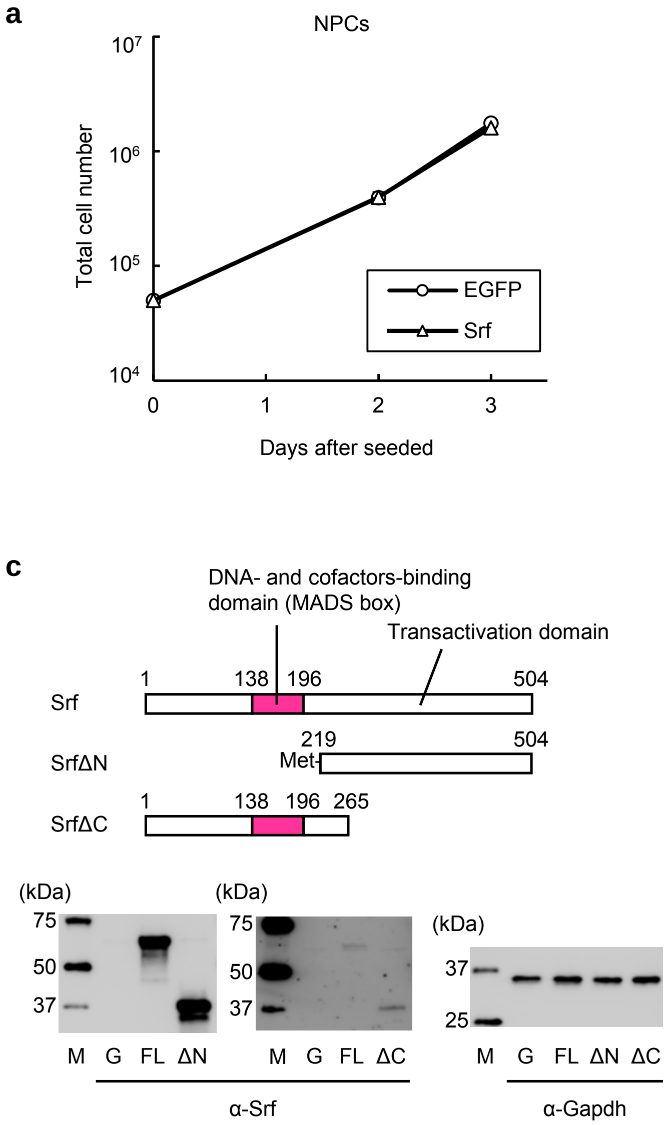

Supplementary Figure 7. Additional information on reprogramming promotion by Srf. (**a**) *Srf* overexpression has little influence on the proliferation of NPCs. Values indicate means ± s.d. (n=3). Vertical axis is log scale. (**b**) Knockdown of *Srf* in NPCs. The expression level of *Srf* mRNA in cells with two different sequences of shRNAs for *Srf* (shSrf) were analyzed by RT-qPCR. Values are means ± s.d. of the *Srf* expression levels normalized to *Gapdh* expression in NPCs relative to the ratio in control cells (n=2, technical duplicate). Dots indicate individual data points. (**c**) Schematic view of the Srf truncation mutants used in Fig. 3a. (Upper panel) Known functional domain structures of full-length Srf, SrfΔN and SrfΔC proteins are shown. Numbers indicate amino acid positions from the N-terminus of full-length Srf. (Lower panel) Immunoblotting of cells expressing EGFP (G) and cells overexpressing full-length Srf (FL), SrfΔN (ΔN) or SrfΔC (ΔC) using anti-Srf antibodies and anti-Gapdh antibody. M, size marker. (**d**) Srf promotes the reprogramming of hepatoblasts. Values are means ± s.d. of the ratio of *Nanog*-positive cells relative to control. Dots indicate individual data points. (**e**) Srf promotes the reprogramming of kidney cells (UBC). Values are means ± s.d. of iPSC colonies relative to control (n=3). Dots indicate individual data points. Asterisks indicate statistically significant differences compared to control (Student's *t*-test; $***P < 0.0005$, $**P < 0.005$).

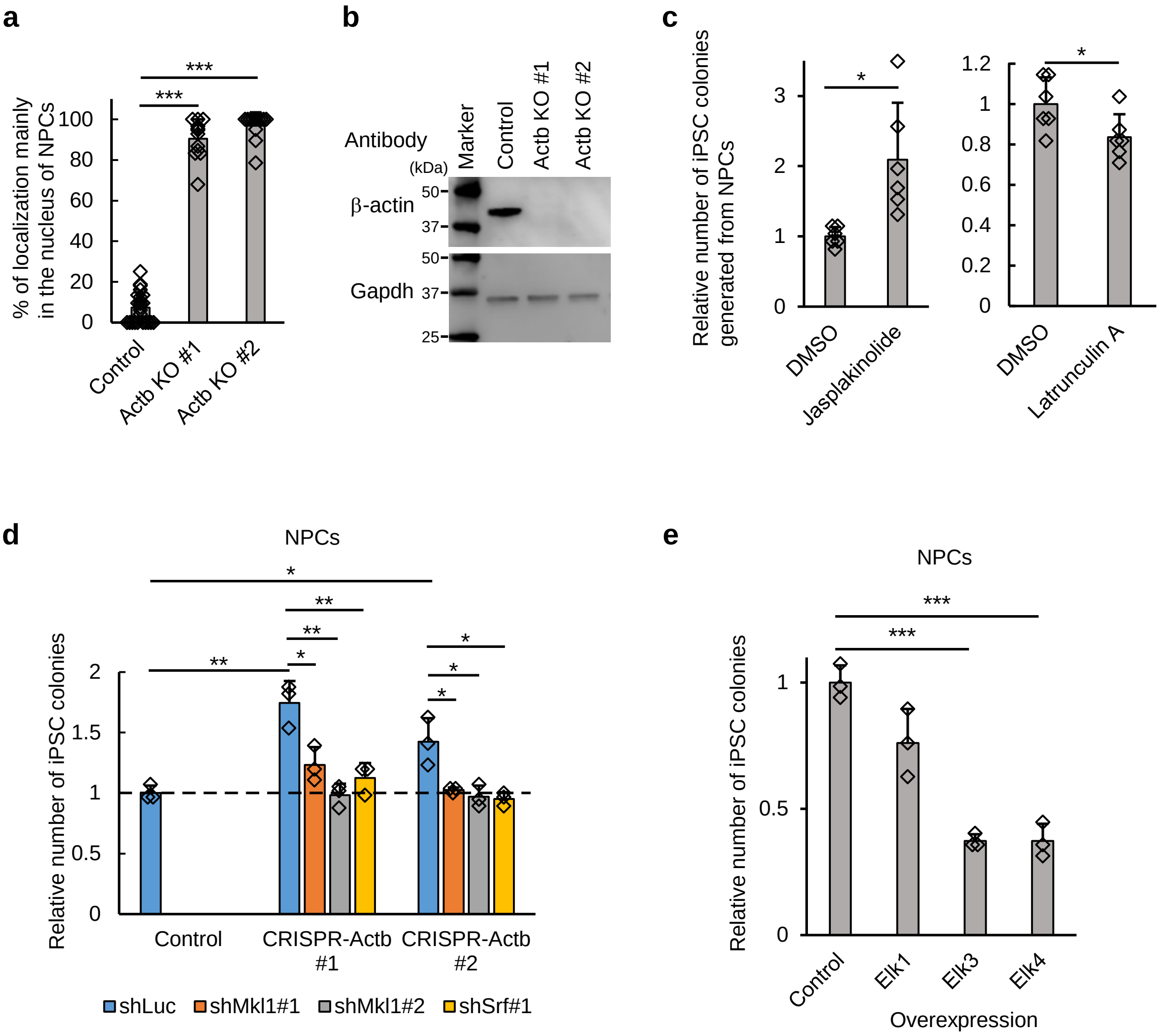

Supplementary Figure 8. Additional information on the effects of Srf cofactors on reprogramming. (**a**) Quantification of NPCs with Mkl1 localization mainly in the nucleus. Values are means ± s.d. of 24 (Control), 12 (*Actb* KO #1) and 12 (*Actb* KO #2) view fields. Student's *t*-test (****P* < 0.0005). **(b)** Immunoblotting using anti-β-actin and Gapdh antibodies. (**c**) Transient exposure to jasplakinolide or latrunculin A at early phase influences the cell reprogramming efficiency. Values are means ± s.d. of iPSC colonies generated from NPCs relative to the control. Data were obtained from samples administered 0.02 to 0.4 µM jasplakinolide (n=6) or latrunculin A (n=6) for 2.5 to 7.5 hrs. Student's *t*-test (**P* < 0.05). (**d**) Knockdown of *Mkl1* or *Srf* attenuates the promoting effect of β-actin depletion on the reprogramming efficiency of NPCs. Values are means ± s.d. of iPSC colonies relative to shLuc-transfected control cells (n=3). (**e**) TCF components inhibit cell reprogramming of NPCs. Values are means ± s.d. of iPSC colonies relative to control (n=3). Dots indicate individual data points. Asterisks indicate statistically significant differences compared to control (two-sided Student's *t*-test; ****P* < 0.0005).

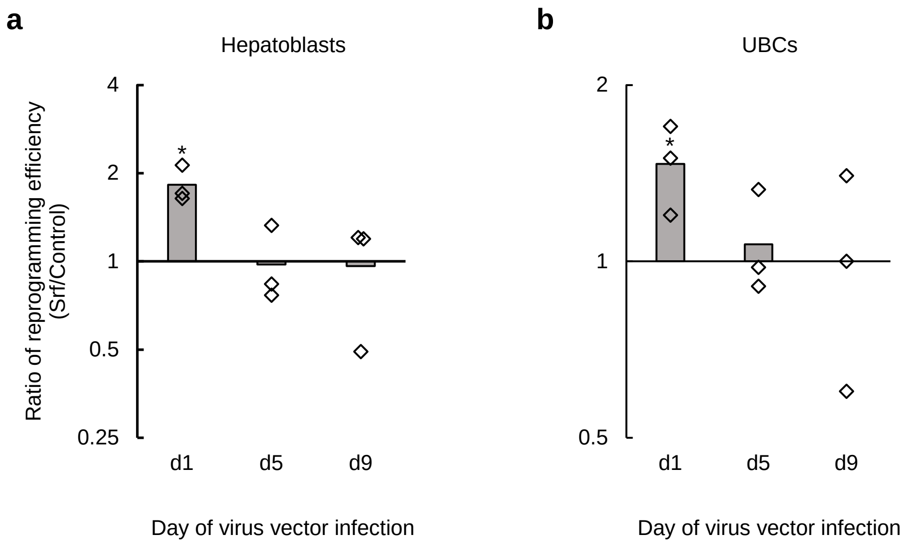

Supplementary Figure 9. Srf promotes reprogramming at early phase in hepatoblasts and UBCs (kidney cells). Hepatoblasts (**a**) and UBCs (**b**) were infected with *egfp* (control)-expressing lentivirus or *Srf*-overexpressing lentivirus at the days indicated (d1 is the day of reprogramming initiation by Dox addition). Vertical axis shows the ratio of the reprogramming efficiency of *Srf*-overexpressing cells to *egfp*-expressing cells in log scale (n=3). Dots indicate individual data points. Asterisks indicate statistically significant differences analyzed by two-sided Student's *t*-test between *Srf* overexpression and control on the day of infection. (*P < 0.05).

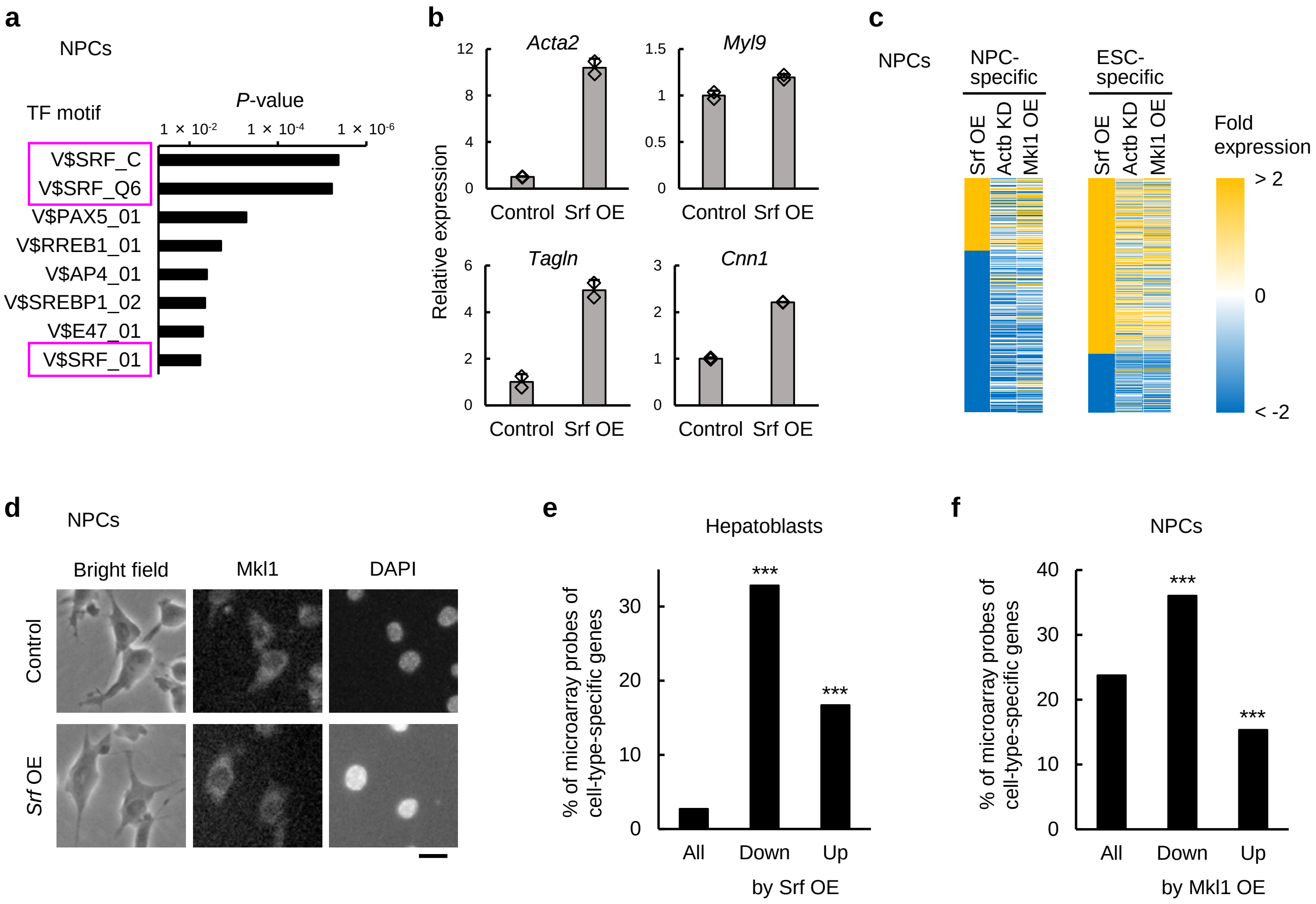

Supplementary Figure 10. Additional information on genome-wide gene expression analyses of Mkl1 and Srf. (**a**) Srf-binding consensus motifs are enriched upstream of genes upregulated in NPCs by *Srf* overexpression. Genes upregulated more than 2-fold were subjected to a motif search using Transfac to search protein-binding consensus sequences that were enriched in upstream regions. (**b**) Overexpression of *Srf* upregulates the expression of *Srf* target genes. Expression levels of Mkl1-mediated Srf targets relative to the expression of each gene in the control cells are shown (n=2, technical duplicate). Values are means ± s.d. of the microarray data. Dots indicate individual data points. (**c**) *Actb* knockdown and *Mkl1* overexpression influence cell-type-specific gene expressions similarly to *Srf* overexpression. A heatmap showing expression changes in NPCs with *Srf* overexpression, *Actb* repression or *Mkl1* overexpression. NPC genes (> 5-fold than in ESCs) and ESC genes (> 5-fold than in NPCs) were selected. Among them, genes whose expressions were changed more than 2-fold by *Srf* overexpression were further selected and used to draw the heatmap. (**d**) Mkl1 localization mainly in the cytoplasm is not altered by *Srf* overexpression. Immunofluorescence microscopy using an anti-Mkl1 antibody and the corresponding bright field and DAPI-staining images of control and *Srf*-overexpressing NPCs. Bar, 20 μm. (**e**) Cell-type-specific genes were enriched in genes downregulated by *Srf* overexpression in hepatoblasts. Percentages of microarray probes of hepatoblast genes in all genes, genes whose expression was downregulated and genes whose expression was upregulated by *Srf* overexpression in hepatoblasts. (**f**) Cell-type-specific genes were enriched in genes downregulated by *Mkl1* overexpression in NPCs. Asterisks indicate significant differences between the indicated group and genes that are not contained in the indicated group (two-sided Fisher's exact test; ***$P < 0.0005$).

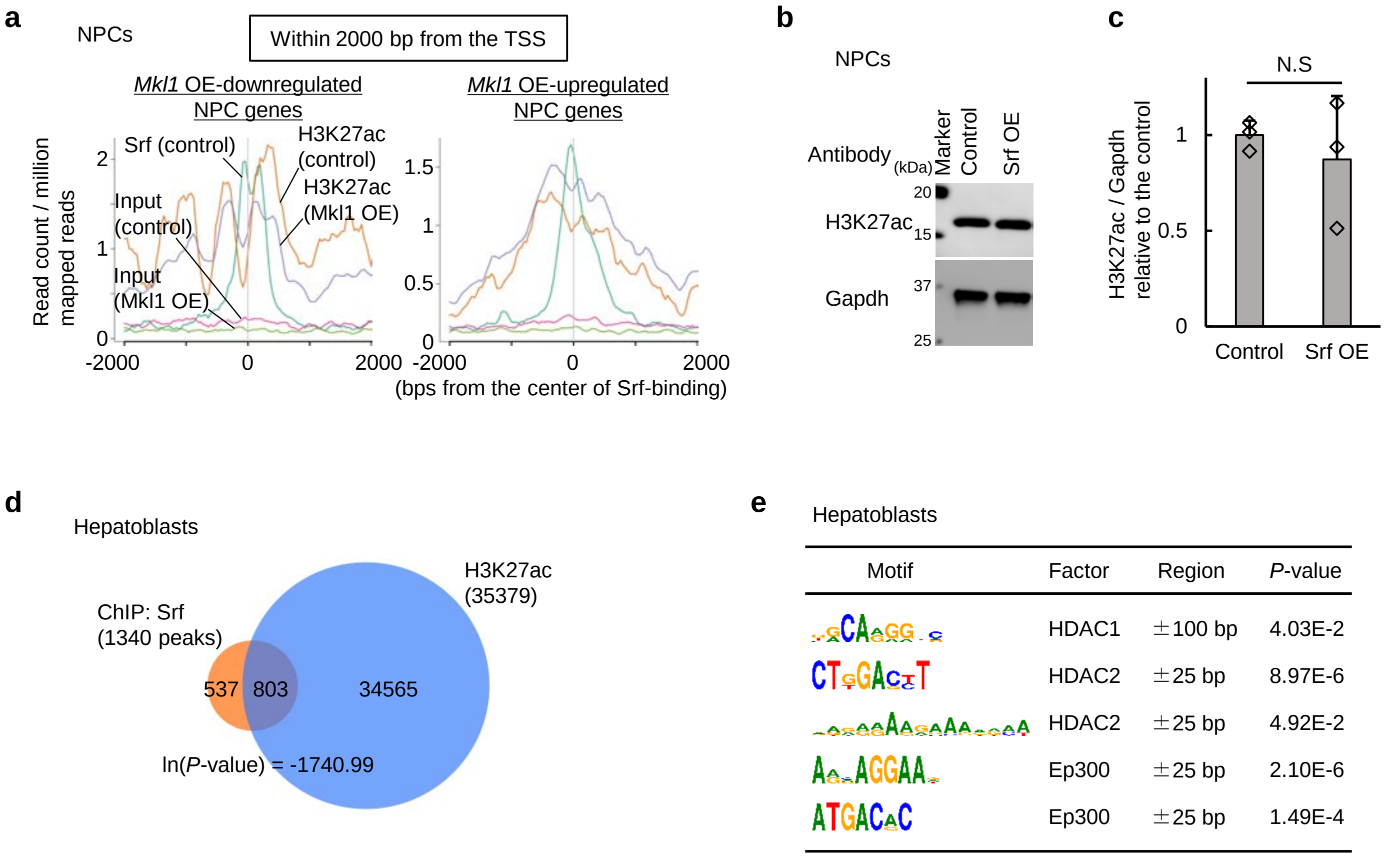

Supplementary Figure 11. Additional information on ChIP-seq analyses. (**a**) H3K27ac marks are decreased by *Mkl1* overexpression around Srf-binding sites near the TSS of cell-type-specific genes in NPCs. Srf-binding and H3K27ac marks in NPCs were analyzed for genomic regions to which Srf was bound within 2000 bp of the TSS in NPC genes that were downregulated or upregulated more than 1.5-fold by the overexpression of *Mkl1*. (**b**) Representative Western blot images of the control and *Srf*-overexpressing NPCs using anti-H3K27ac and anti-Gapdh antibodies. (**c**) The global level of H3K27ac was not significantly altered by *Srf* overexpression. Quantitative analysis of the band reacted with an anti-H3K27ac antibody. Values are means ± s.d. of the band intensities estimated by ImageJ and normalized to Gapdh (control is set to 1) (n=3). Dots indicate individual data points. N.S, no significant difference by two-sided Student's *t*-test. (**d**) Srf preferentially binds to H3K27ac-marked genomic regions in hepatoblasts. A Venn diagram showing the overlap between Srf-binding sites and H3K27ac-marked regions determined by ChIP-seq. The peak number in the overlapped area is based on the number of Srf-binding peaks. Overlap was analyzed by the hypergeometric test. (**e**) Matrices of consensus binding motifs of HDACs and Ep300 that are enriched near Srf-binding sites in hepatoblasts. A motif analysis of ChIP-seq data of Srf in hepatoblasts indicated that the matrices of motifs of HDAC1, HDAC2 and Ep300 were enriched within 100 bp (HDAC1) or 25 bp (HDAC2 and Ep300) of Srf-binding sites. Fisher's exact test.

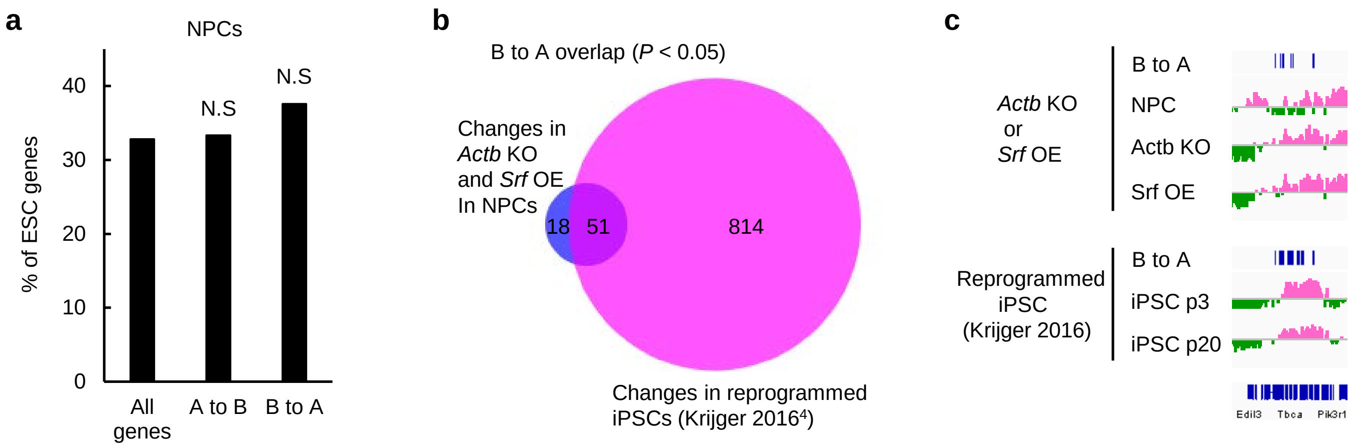

Supplementary Figure 12. Additional information on Hi-C analyses. (**a**) ESC genes are not enriched in genes that change their subnuclear compartment from A to B or in those from B to A by the β-actin-Srf pathway. Percentages of ESC genes in all genes and genes whose compartment was changed by the β-actin-Srf pathway from A to B and from B to A in NPCs. N.S, no significant difference between the indicated group and genes that are not contained in the indicated group (two-sided Fisher's exact test). (**b**) Chromatin organization changes by the β-actin-Srf pathway partially resemble the case of reprogramming to pluripotency[4]. A Venn diagram of peak numbers of chromatin organization changes from compartment B to compartment A shows that more than 70% of changes by the β-actin-Srf pathway were overlapped with changes by reprogramming to pluripotency. (**c**) A region on chromosome 13 where the patterns from B to A were similar in *Actb* knockout, *Srf* overexpression and reprogramming to iPSCs.

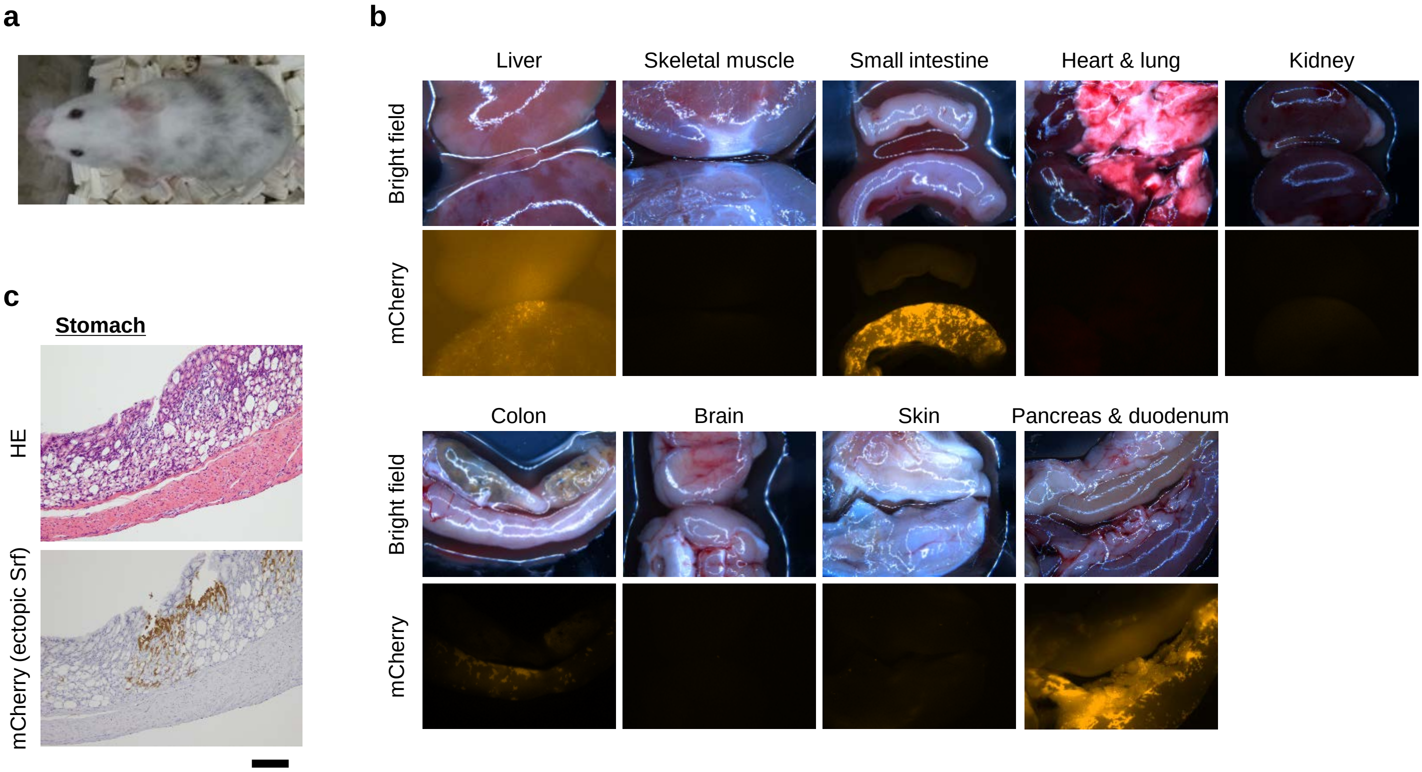

Supplementary Figure 13. Additional information on chimeric mice overexpressing *Srf*. (**a**) A representative coat color of a KH2-Srf chimeric mouse subjected to phenotypic analyses of *Srf* overexpression. (**b**) Ectopic expression of the *Srf* transgene is detected in various tissues of chimeric mice. Images show tissues from chimeric mice treated (lower) or not treated (upper) with Dox for 4 days put side by side. (**c**) Srf misactivation causes dysplasia-like alterations in the epithelia of stomach. Images of HE staining (upper panel) and immunohistochemistry for a roughly corresponding view field (lower panel) are shown. Bar, 100 µm.

a

Microarray data

- Ulcerative colitis (UC)
  - Sigmoid colon of patients (n=25) vs healthy donors (n=24)
- Type 1 diabetes (T1D)
  - T helper cells of patients (n=4) vs healthy donors (n=4)
- Alzheimer's disease (AD)
  - Hippocampus of patients (n=7) vs healthy donors (n=10)
- Multiple sclerosis (MS)
  - T helper cells of patients (n=4) vs healthy donors (n=4)
- Atrial fibrillation (AF)
  - Atrial cardium with patients (n=10) vs healthy donors(n=20)

Downregulated genes →

P-value: < 0.00005 · 0.05 · > 0.05

TF motifs	UC	T1D	AD	MS	AF
PBX1	1.68E-02				
SRF	1.91E-02	5.72E-04	8.89E-03	1.36E-02	1.58E-03
NFKAPPAB50	2.01E-02	1.45E-04			
FREAC3	2.04E-02				
STAF	4.12E-02				
HNF1		6.02E-04			
VMAF		6.84E-04			
ISRE		1.29E-03			
NFKB		8.33E-03			
CREB		8.97E-03			3.38E-03
ATF		9.45E-03			
CAAT		1.50E-02			5.36E-03
AP1		1.60E-02			
MEF2		2.12E-02			3.88E-03
SOX9		2.17E-02			
P53		2.89E-02			
HEN1		2.97E-02			
TAXCREB		3.05E-02			5.51E-04
BRACH		3.33E-02			
CREBP1		4.03E-02			2.77E-03
MEIS1AHOXA9		4.34E-02			
NFY		4.66E-02		8.16E-03	
NFKAPPAB		4.88E-02			
NRSF			4.32E-08		
RREB1			2.75E-03		
EGR2			1.01E-02		
EGR3			1.10E-02		
T3R			2.97E-02		3.85E-02

TF motifs	UC	T1D	AD	MS	AF
HNF4			3.82E-02		
PPARA			3.98E-02		
IK3			4.27E-02		
RORA1				7.09E-04	
FREAC4				3.12E-03	
PAX3				3.69E-03	6.87E-03
XFD3				5.58E-03	
CREBP1CJUN				1.22E-02	2.77E-02
FREAC2				1.82E-02	
MYOGNF1				2.19E-02	
GATA2				3.47E-02	
NFE2				3.64E-02	
CDP				4.04E-02	
RSRFC4				4.15E-02	1.91E-02
E2F				4.91E-02	8.35E-04
EGR1					2.99E-05
VMYB					1.21E-03
CDPCR1					5.59E-03
E4BP4					9.04E-03
ELK1					1.01E-02
IRF2					1.06E-02
USF					1.09E-02
EVI1					2.08E-02
HLF					2.72E-02
R					4.04E-02
GATA3					4.21E-02
PAX5					4.61E-02
BRN2					4.90E-02

b

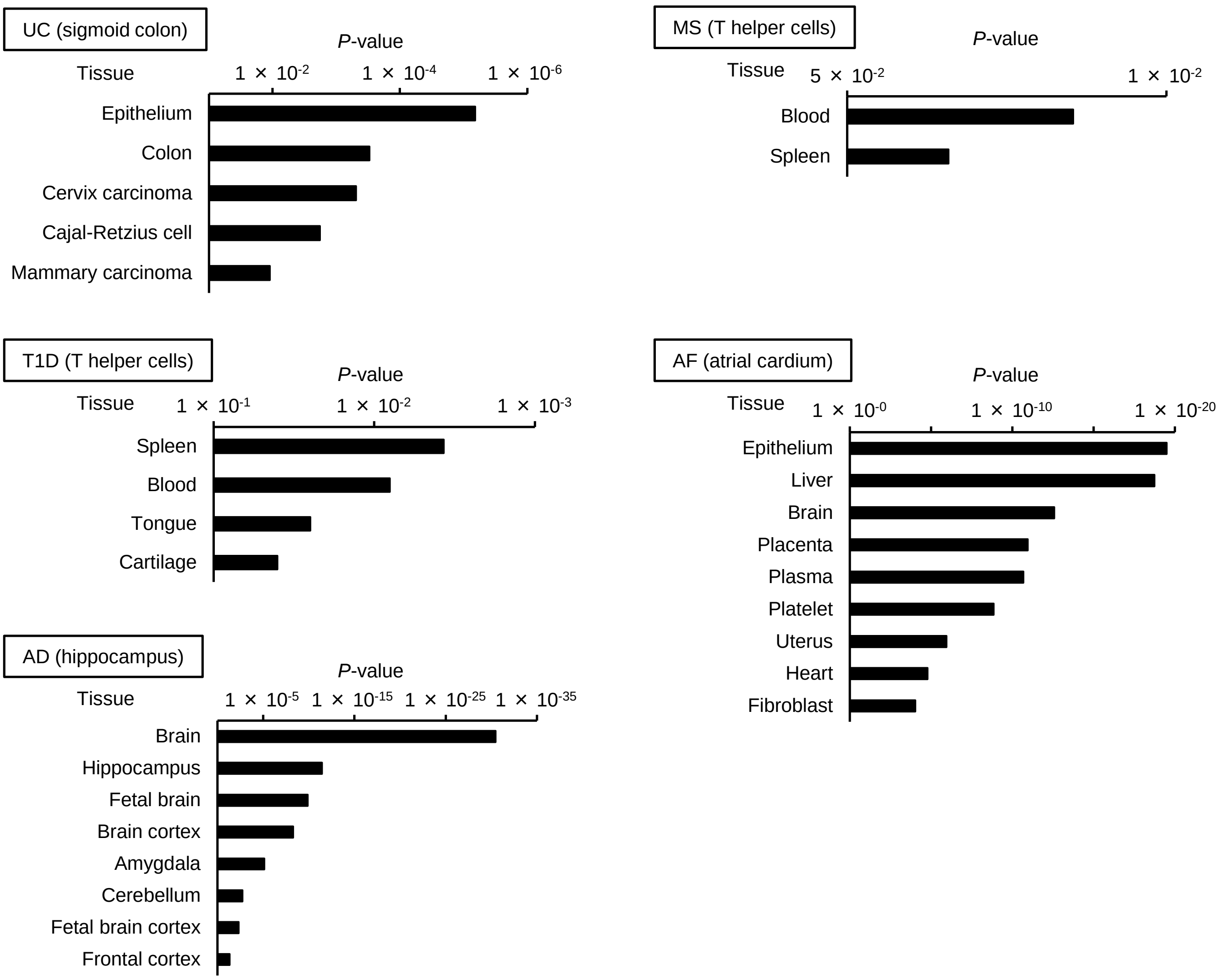

Supplementary Figure 14. Additional information on Srf misactivation causing diseases. (**a**) TF motif analyses of genome-wide expression data of various diseases. Public microarray data of the sigmoid colon of ulcerative colitis (UC) patients (GSE11223), Th cells of type 1 diabetes (T1D) patients (GSE60424), the hippocampus of Alzheimer's disease (AD) patients (GSE36980), Th cells of multiple sclerosis (MS) patients (GSE16461), and the atrial cardium of atrial fibrillation (AF) patients (GSE2240) were re-analyzed, and regions upstream of genes downregulated in these patients were subjected to motif search analyses. Motifs with significant enrichment ($P < 0.05$, Fisher's exact test) in downregulated genes are shown. The thresholds of downregulation are 1.4× for UC, 1.5× for T1D, AD and MS, and 1.2× for AF. (**b**) Cell-type-related genes are enriched in genes downregulated in various diseased cells. Tissue expression analyses[1,2] of genes downregulated (< 0.77× for UC, < 0.67× for T1D, AD and MS, and < 0.83× for AF) in the indicated cells. Fisher's exact test.

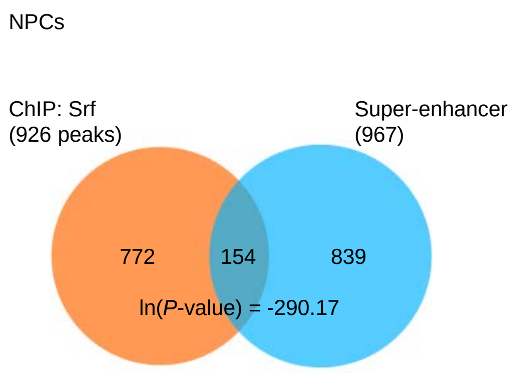

Supplementary Figure 15. A significant portion of Srf-binding sites are on super-enhancers. Venn diagram showing overlaps of Srf-binding sites determined by ChIP-seq and super-enhancer regions predicted by ROSE[5-7] in NPCs. The peak number shown in the overlapped area is based on the number of Srf-binding peaks. The overlap was analyzed by the hypergeometric test.

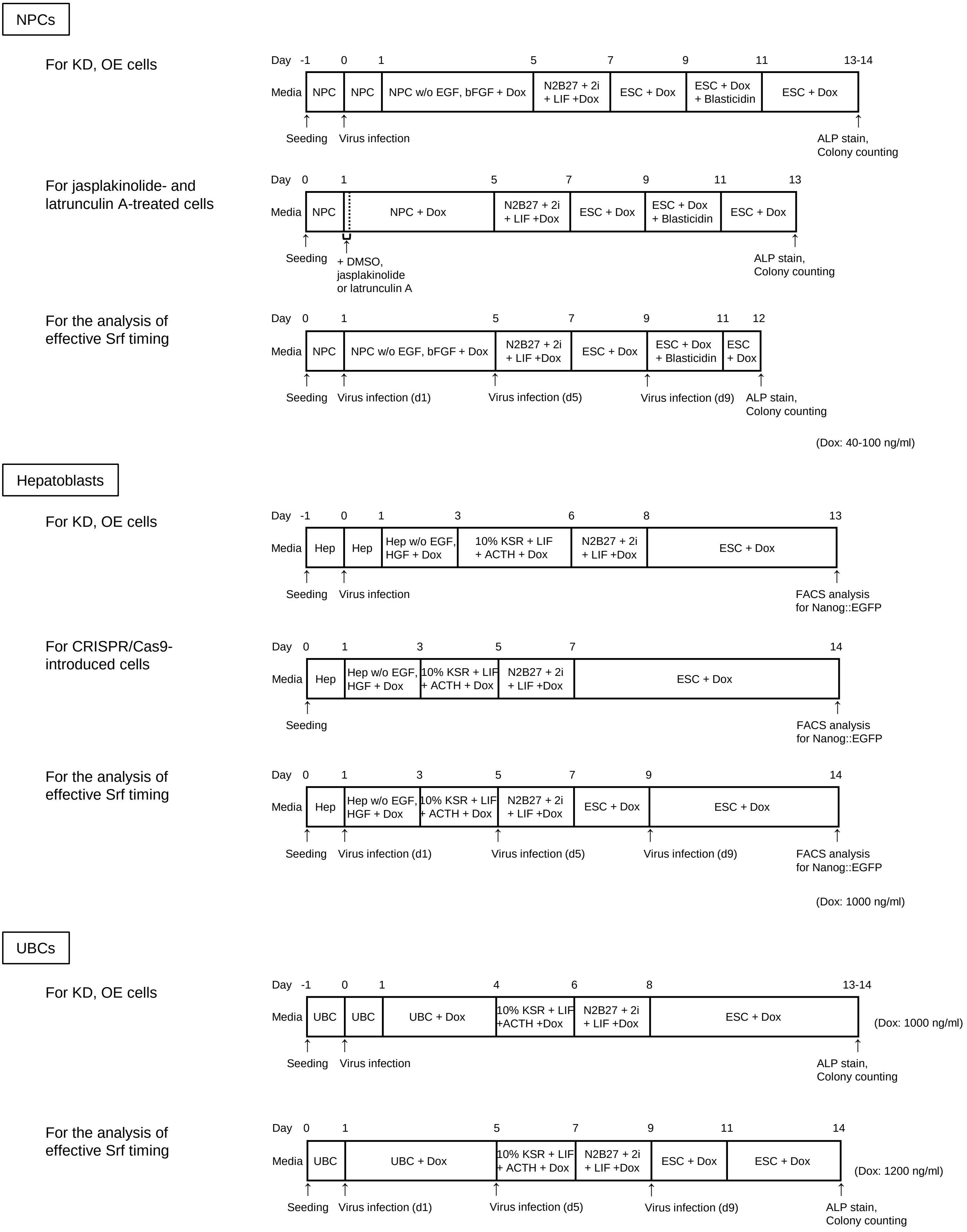

Supplementary Figure 16. Culture conditions of reprogramming used in this study. Reprogramming was performed by cultures as shown in this figure unless stated otherwise in the text.

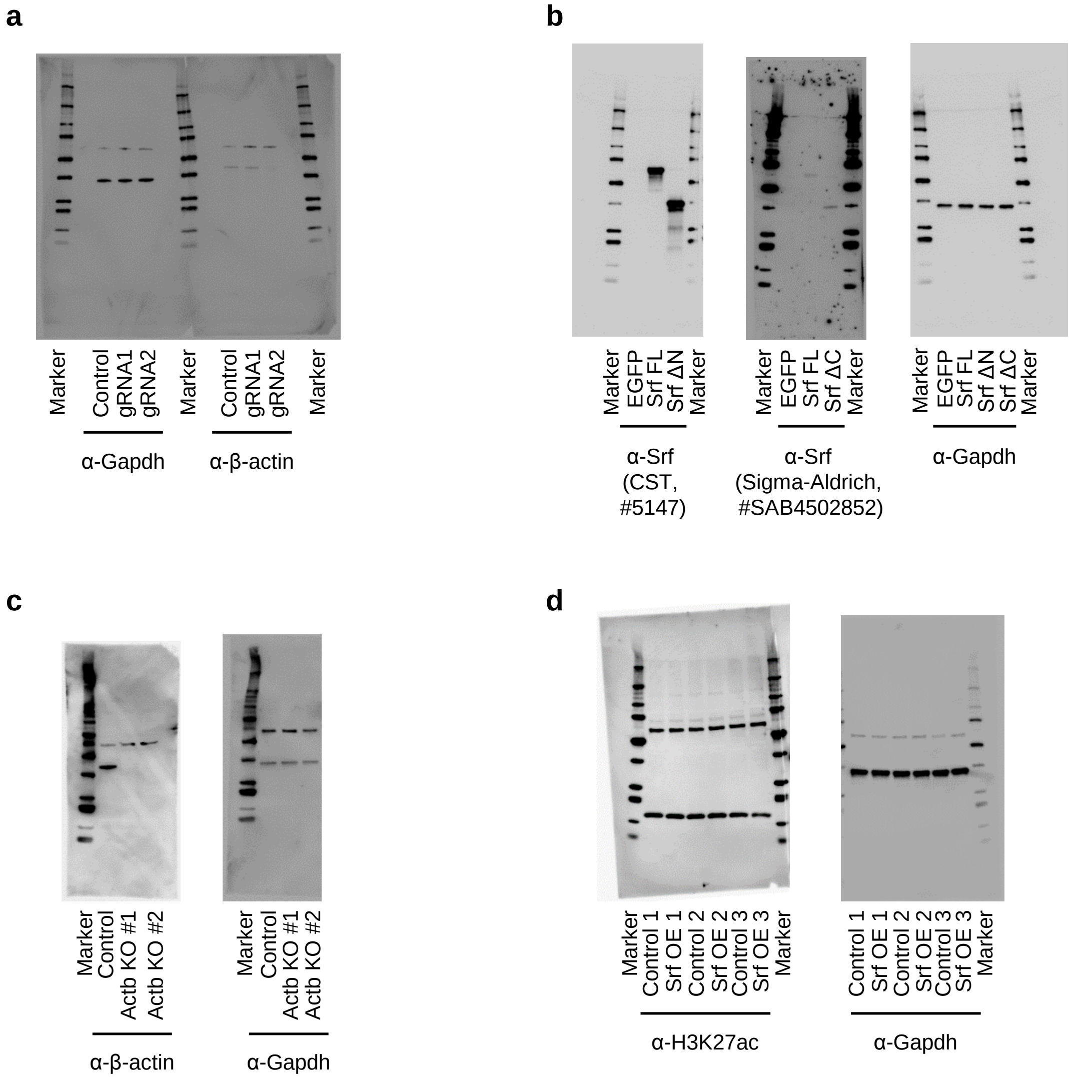

Supplementary Figure 17. Full images of Western blot. Full blot images of Supplementary Fig. 6b (**a**), Supplementary Fig. 7c (**b**), Supplementary Fig. 8b (**c**) and Supplementary Fig. 11b (**d**). Lanes irrelevant to this study at the right side are cut off from the image of Gapdh in c.

Supplementary References

1 Huang da, W., Sherman, B. T. & Lempicki, R. A. Bioinformatics enrichment tools: paths toward the comprehensive functional analysis of large gene lists. *Nucleic acids research* **37**, 1-13 (2009).

2 Huang da, W., Sherman, B. T. & Lempicki, R. A. Systematic and integrative analysis of large gene lists using DAVID bioinformatics resources. *Nature protocols* **4**, 44-57 (2009).

3 Yang, C. S., Chang, K. Y. & Rana, T. M. Genome-wide functional analysis reveals factors needed at the transition steps of induced reprogramming. *Cell Rep* **8**, 327-337 (2014).

4 Krijger, P. H. *et al*. Cell-of-Origin-Specific 3D Genome Structure Acquired during Somatic Cell Reprogramming. *Cell stem cell* **18**, 597-610 (2016).

5 Whyte, W. A. *et al.* Master transcription factors and mediator establish super-enhancers at key cell identity genes. *Cell* **153**, 307-319 (2013).

6 Loven, J. *et al.* Selective inhibition of tumor oncogenes by disruption of super-enhancers. *Cell* **153**, 320-334 (2013).

7 Hnisz, D. *et al.* Super-enhancers in the control of cell identity and disease. *Cell* **155**, 934-947 (2013).
