# Supplementary material for: Srf destabilizes cellular identity by suppressing cell-type-specific gene expression programs
Source: Nat Commun. 2018 Apr 11;9:1387. doi: 10.1038/s41467-018-03748-1 (PMC5895821; doi:10.1038/s41467-018-03748-1)
Supplement: Supplementary file 2 — Description of Additional Supplementary Files(PDF 4 kb) [file 41467_2018_3748_MOESM2_ESM.pdf]

Description of Additional Supplementary Files

File Name: Supplementary Data 1
Description: shRNA targets enriched in the screenings of NPCs

File Name: Supplementary Data 2
Description: shRNA targets enriched in the screening of hepatoblasts

File Name: Supplementary Data 3
Description: Overlapped shRNA targets among the screenings of MEFs, NPCs and hepatoblasts
